# Lifestyle as well as metabolic syndrome and non-alcoholic fatty liver disease: an umbrella review of evidence from observational studies and randomized controlled trials

**DOI:** 10.1186/s12902-022-01015-5

**Published:** 2022-04-10

**Authors:** Xiaojuan Peng, Juan Li, Hailiang Zhao, Junlong Lai, Junqin Lin, Shaohui Tang

**Affiliations:** 1 Department of Endocrinology, Liuzhou People’s Hospital, Liuzhou, Guangxi 545006 PR China; 2 Department of Gastroenterology, The First Affiliated Hospital, Jinan University, Guangzhou, Guangdong 510630 PR China; 3 Department of Infectious Disease, The Third Affiliated Hospital of Jinzhou Medical University, Jinzhou, 121001 PR China

**Keywords:** Lifestyle, Metabolic syndrome, non-alcoholic fatty liver disease, Umbrella review, Meta-analyses

## Abstract

**Background & Aims:**

Recent epidemiological studies have indicated that NAFLD is pathologically associated with a sedentary lifestyle, unhealthy dietary habits and metabolic syndrome. An umbrella review of meta-analyses was performed to summarize the quality of evidence regarding the epidemiologic associations between lifestyle, metabolic syndrome, and non-alcoholic fatty liver disease (NAFLD) in regards to risk and treatment.

**Methods:**

We searched PubMed, Web of Science and Embase Database from inception until June 1, 2021. Meta-analyses of observational studies and randomized controlled trials (RCTs) examining the associations of lifestyle as well as metabolic syndrome with NAFLD risk or treatment were screened. We assessed meta-analyses of observational studies based on random-effect summary effect sizes and their *P* values, 95% prediction intervals, heterogeneity, and small-study effects. For meta-analyses of RCTs, outcomes with a random-effect *P* < 0.005 and a high-GRADE assessment were classified as strong evidence.

**Results:**

A total of 37 publications were included in this review: twenty-two publications reporting 41 meta-analyses of observational studies (37 unique outcomes) and 15 publications reporting 81 meta-analyses of RCTs (63 unique outcomes) met the inclusion criteria. Methodological quality was high for 97% of the included meta-analyses. Quality of evidence was rated high only for the association of sugar-sweetened soda consumption with increased NAFLD risk in meta-analyses of observational studies. Only 3 therapeutic interventions (green tea improving ALT, TG, TC and LDL, omega-3 PUFAs improving HOMR-IR and plasma glucose, and exercise improving RT and ALT) from meta -analyses of RCTs with suggestive (change to high/low/etc) levels of evidence were identified.

**Conclusion:**

Despite many meta-analyses exploring the associations of lifestyle as well as metabolic syndrome with the risk or treatment of NAFLD, robust clinical RCTs are needed to further investigate the associations between lifestyle modifications and incidence of NAFLD or therapeutic effects on disease progression.

**Supplementary Information:**

The online version contains supplementary material available at 10.1186/s12902-022-01015-5.

## INTRODUCTION

Nonalcoholic fatty liver disease (NAFLD) encompasses a spectrum of liver diseases ranging from non-alcoholic hepatic steatosis and non-alcoholic steatohepatitis (NASH) that can further progress to cirrhosis and hepatocellular carcinoma (HCC) [1, 2]. NAFLD has become the most common cause of chronic liver disease worldwide, with a global prevalence of 22–29% in adults worldwide [3]. More and more literature is growing to support that NAFLD is a manifestation of metabolic syndrome (central adiposity, dyslipidemia, hyperglycemia, hypertension, and hyperuricemia), with insulin resistance perhaps being the common pathogenic event [4–6]. Weight gain and the presence of metabolic syndrome remain the strongest risk factors for the development of NAFLD [3, 7, 8]. On the other hand, the prevalence of NAFLD is carried with a higher risk of type 2 diabetes mellitus [9], cardio-metabolic and other liver-related complications [10]. Therefore, NAFLD is emerging as a major threat to general health.

Recent epidemiological studies have indicated that NAFLD is pathologically associated with a sedentary lifestyle, unhealthy dietary habit and metabolic syndrome [2, 11–13]. Many published meta-analyses have shown that smoking, short sleep duration, red meat, soft drinks, sugar (glucose and fructose), obesity, and hyperuricemia appear to increase the risk of NAFLD [3, 14–18]. Inversely, coffee, green tea, modest alcohol, nuts, exercise, and weight loss are reported to have a decreased risk of developing NAFLD [15, 19–22]. Currently, lifestyle changes and exercise represent the first-line therapy for NAFLD, because pharmacological agents have been limited by realistic concerns related to effectiveness and safety, and no medical intervention has been approved for treating NAFLD in clinical practice [23, 24]. Several meta-analyses have reported that green tea, coffee, low carbohydrate diet, omega-3, exercise, and weight loss, were a proven treatment for NAFLD [22, 25–29].

Although several systematic reviews and meta-analyses have examined associations between lifestyle or metabolic syndrome and NAFLD, there has been no existing umbrella reviews to summarize and critically appraise this body of evidence until June, 2021. Therefore, this study aimed to perform an umbrella review to gain a strength and validity of the evidence derived from systematic reviews and meta-analyses of the association between lifestyle as well as metabolic syndrome and NAFLD.

## METHODS

Our protocol has been registered in PROSPERO (CRD42020186604). The systematic literature search was conducted according to the preferred reporting items for systematic reviews and meta-analyses (PRISMA) guidelines [30].

### Literature search

For this umbrella review, we searched PubMed, Web of Science and Embase Database.

for meta-analyses about associations between lifestyle or metabolic syndrome and the risk or treatment of NAFLD from inception until June 1, 2021. The search terms were (lifestyle or exercise or dietary or diet or training or behavior or nutrition or sport or physical activity or weight reduction or weight loss or energy restriction) or (metabolic syndrome or obesity, central obesity, WHR, BMI, hyperglycemia, hypertension, hyperuricemia, serum uric acid) AND (NAFLD or non-alcoholic fatty liver or nonalcoholic fatty liver or non-alcoholic steatohepatitis or nonalcoholic steatohepatitis or non-alcoholic steatosis or nonalcoholic steatosis or non-alcoholic liver steatosis or nonalcoholic liver steatosis or non-alcoholic hepatic steatosis or nonalcoholic hepatic steatosis) AND (systematic review or meta-analysis). We also carried out a manual screen of the references of eligible articles. The search was independently performed by three investigators (X.P., J.L., and H.Z.) and any differences in the literature search were resolved through consensus.

### Selection of meta-analyses

Studies were included if they met the following criteria: (1) Studies included meta-analysis of randomized controlled trials (RCTs) and/or observational studies; (2) Studies considered the incidence or treatment of NAFLD as the outcome; (3) Studies investigated the associations between different lifestyles or metabolic syndrome and incidence or treatment of NAFLD. Review articles without quantitative statistical analysis, RCTs including animal trials or in vitro studies, and studies on genetic polymorphisms related to lifestyle or metabolic syndrome and the risk or treatment of NAFLD were excluded. Children were excluded. Articles that were not published in English were also excluded. If a single meta-analysis was divided into cohort and case-control studies without a total estimated effect size that included both, we reported the results of the cohort study as it was less affected by recall and selection biases.

### Data extraction

One author (X.P.) extracted data, which was separately checked by the other authors (J.L. and H.Z.). From each eligible meta-analysis on observational studies or RCTs, the following information was extracted: first author and publication year, outcome, number of studies included, total population, number of cases, measure of exposure, effect sizes (risk ratio (RR), odds ratio (OR), hazard ratio (HR), mean difference (MD), standardized mean difference (SMD), weighted mean difference (WMD), and 95% confidence intervals), and any reported estimate of heterogeneity. Finally, the type of effect model, publication bias by Egger’s test, and dose-response analyses were abstracted when possible. When overlapping meta-analyses were published on the same association, we included the one with the most recent and the largest number of disease cases. In a few exceptions where the most recent was not the largest meta-analysis, we examined the reason for this discrepancy. If the most recent included prospective studies and the largest one had fewer prospective studies plus some retrospective data, we kept the one with the largest amount of prospective data; otherwise we kept the largest meta-analysis. If a high-versus-low meta-analysis as well as a dose-response meta-analysis was available for one exposure or treatment, we presented the dose-response meta-analysis. Any discrepancies in the extracted data were resolved with discussion.

### Assessment of methodological quality

The eleven items of Assessment of Multiple Systematic Reviews (AMSTAR) checklist were performed to evaluate reporting and methodological quality of all included systematic reviews and meta-analyses [31]. Each question can be answered with “yes,” “no,” “can’t answer,” and “not applicable.” A “yes” scores one point, whereas the other answers score 0 points. An overall score of at least 8 points was defined as the cutoff value for high quality, 4–7 points as moderate quality, and 3 points or less as low quality (Supplementary Table 1).

### Evaluation of the grading of evidence

We classified evidence from meta-analyses of observational studies with nominally statistically significant summary results into three categories (high, moderate, and low) [32]. The strength of epidemiologic evidence was assessed according to the following criteria [33–35]: (1) precision of the estimate (ie, *P* < .001 [36, 37], a threshold associated with significantly fewer false positive results, and more than 1000 cases of the disease), (2) consistency of results (I^2^ < 50%; Cochran Q test, *P* > .10), and (3) no evidence of small-study effects (*P* > .10). The strength of the epidemiologic evidence was rated as high (when all of these criteria were satisfied), moderate (if a maximum of 1 criterion was not satisfied and *P* < .001 was found), or weak in all other cases (*P* < .05). Whenever the *P* value was not reported, it was calculated from the 95% confidence interval of the pooled effect estimate by using a standard method [38]. Evidence from meta-analyses of RCTs was assessed in the light of the significance of the summary effect (*P*  <.01, .01 ≤ *P* < .05, *P*  ≥.05), presence of large heterogeneity (I^2^ > 50%), and small study effects(*P* > .10).

### Data analysis

For each meta-analysis, we extracted the summary effect size and its 95% confidence intervals (CI) through random-effects models. Whenever a fixed effect model was originally used, we recalculated the summary effect sizes and corresponding 95% CI by using the random effect model. We tested for evidence of small-study effects using the Egger’s regression asymmetry test to investigate if smaller studies yielded larger effect sizes compared with larger studies (significance threshold *P* < .10) [39]. All the analyses were conducted with STATA 13.0 (STATA Corp, Texas, USA). For all tests (except for heterogeneity and small-study effects), *P* < .05 was considered statistically significant.

## RESULTS

### Characteristics of Meta-Analyses

**Fig. 1:**
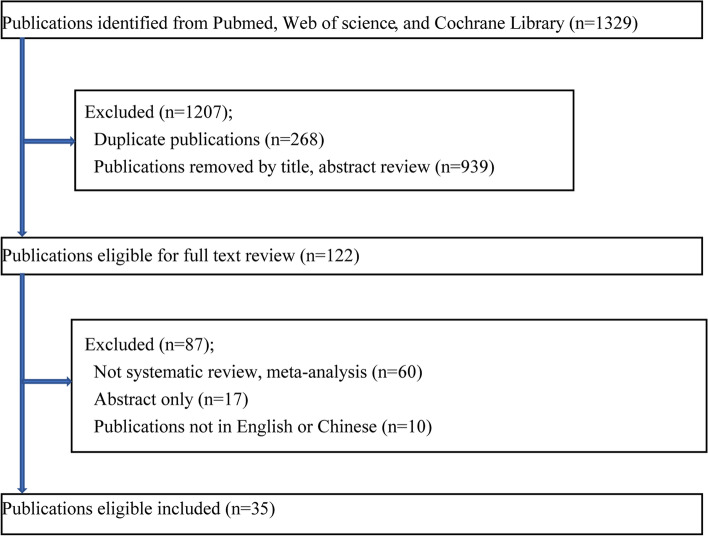
Flow diagram of literature search and study selection

The search strategy found 1329 publications, as shown in (Fig. 1). The umbrella review identified 35 publications with 122 meta-analysis results, of which 22 publications [15, 16, 20, 21, 26, 29, 40–55] reported 41 meta-analyses of observational studies and 15 publications [22, 25–29, 56–64] reported 81 meta-analyses of RCTs. In the 41 meta-analyses of observational studies, 4 meta-analyses showed overlapping results that were removed (Supplementary Table 2). Of the 81 meta-analyses of RCTs, 18 similarly showed overlapping results and were therefore removed. Eventually**,** 100 unique meta-analyses were retained (37 meta-analyses of observational studies (Supplementary Table 3) and 63 meta-analyses of RCTs (Supplementary Table 4). The median number of studies included in meta-analyses of observational studies was 5 (range 2–21), the median number of participants was 6177 (73–381,655), and the median number of cases was 2810 (41–20,149). The median number of studies included in meta-analyses of RCTs was 8 (range 2–21**)**, the median number of participants was 502 (61–13,426), and the median number of cases was 122 (11–1496).

### Quality assessment of meta-analyses

The AMSTAR rating for all studies was determined to be high for 97% or moderate for 3% (Supplementary Table 5). The most common reasons for downgrading quality were absence of a registered protocol, non-satisfactory reporting/evaluation of the risk of bias in primary studies, and inappropriate methodology.

### Risk of NAFLD

#### Factors that increase the risk of NAFLD

**Fig. 2:**
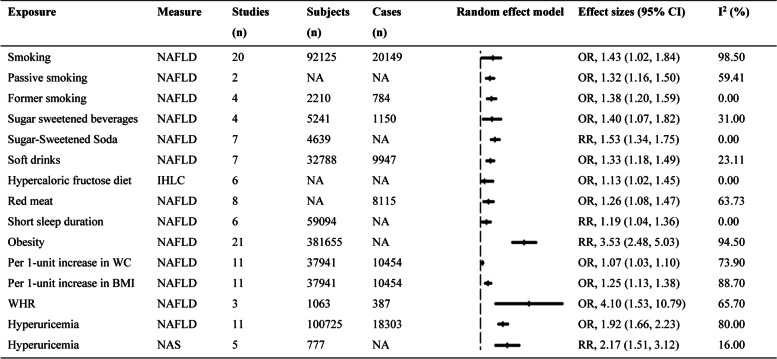
The factors that increased the risk of NAFLD. NAFLD, non-alcoholic fatty liver disease; CI, confidence interval; OR, odds ratio; RR, risk ratio; NA, not available

The 15 factors that increased the risk of NAFLD were presented below (Fig. 2). Compared with non-smoking, smoking, passive smoking, and former smoking increased the risk of NAFLD by about 1.43-fold (OR, 1.43; 1.02, 1.84), 1.32-fold (OR, 1.32; 1.16, 1.50), and 1.38-fold (OR, 1.38; 1.20, 1.59), respectively [16]. On the other hand, consumption of sugar sweetened beverages, sugar sweetened soda, and soft drinks were significantly associated with a increased risk of NAFLD ((OR,1.40; 1.07, 1.82), (RR, 1.53; 1.34, 1.75), and (OR, 1.33; 1.18, 1.49), respectively) [40–42]; compared with the consumption of a weight-maintenance diet, hypercaloric fructose diet intake significantly increased intrahepatic lipid content (IHLC) (OR, 1.13; 1.02, 1.45) in healthy male adults [43]. Red meat was significantly associated with an increased risk of NAFLD (OR, 1.26; 1.08, 1.47) [42]. Furthermore, compared with long sleep duration, short sleep duration was associated with an increased risk of NAFLD (RR, 1.19; 1.04, 1.36) [15]. Obesity increased the risk of developing NAFLD (RR, 3.53; 2.48, 5.03); central obesity posed a greater threat to national health than general obesity, and the summary OR values per-unit increase in waist circumference (WC) and BMI for NAFLD formation were 1.07 (1.03, 1.10) and 1.25 (1.13, 1.38), respectively. In addition, the pooled OR in waist-to-hip ratio (WHR) in relation to NAFLD risk was 4.10 (1.53, 10.79) [45, 54]. Compared to the lowest group, the risk of NAFLD was increased by almost 2-fold (OR, 1.92; 1.66, 2.23) in the highest serum uric acid group [46]; additionally, compared to no hyperuricemia, hyperuricemia was associated with a higher of NAFLD activity score (NAS) (RR, 2.17; 1.51, 3.12) [55].

#### Factors that decrease the risk of NAFLD

**Fig. 3:**
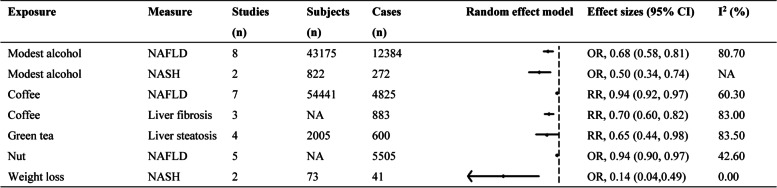
The factors that decreased the risk of NAFLD. NAFLD, non-alcoholic fatty liver disease; CI, confidence interval; OR, odds ratio; RR, risk ratio; NA, not available

The 7 factors that decreased the risk of NAFLD were presented in (Fig. 3). Modest intake of alcohol (for an intake of less than 40 g/day *v* no consumption) decreased the risk of NAFLD (OR, 0.68; 0.58, 0.81) [21]; moreover, modest intake of alcohol was found to have a significant protective effect on the development of non-alcoholic steatohepatitis (NASH) (OR, 0.50; 0.34, 0.74) without any evidence of heterogeneity (*P* > 0.1, I^2^ = 0), and the data were from 822 patients (550 non-drinkers and 272 modest drinkers) diagnosed by liver biopsy [21]. High intake of coffee (more than 3 cups every day) decreased the risk of NAFLD (RR, 0.94; 0.92, 0.97) [47]; compared to the subjects who did not drink coffee, coffee intake decreased the risk of liver fibrosis among NAFLD patients (RR, 0.70; 0.60, 0.82) [48]. Green tea also significantly reduced the risk of NAFLD (RR, 0.65; 0.44, 0.98) [49]. A negative association of nut intake with the possibility of NAFLD was observed (OR, 0.94; 0.90, 0.97) [42]. Weight loss decreased the risk of NASH (OR, 0.14; 0.04**,** 0.49) [29].

#### Factors that are not associated with the risk of NAFLD

The 15 factors that had no significant effects on NAFLD were presented in (Supplementary Table 3). No evidence of associations between current smoking, light smoking, heavy smoking, whole grains, refined grains, fish, fruits, vegetables, eggs, dairy, or legumes and NAFLD was found in the included meta-analyses [16, 42]. Besides, hypercaloric fructose diet did not affect ALT level compared with consumption of a weight-maintaining diet in healthy subjects [44]. Caffeine consumption was not significantly associated with the prevalence of NAFLD [26]. Low carbohydrate diet was not significantly associated with the improvement of ALT and AST level in NAFLD [50].

### Treatment of NAFLD

#### Therapies that improve NAFLD

##### Caffeine

Total caffeine consumption reduced hepatic fibrosis in patients with NAFLD (MD, −91.35; −139.42, −43.27) [26] (Table 1).

**Table 1:** Characteristics of 48 meta-analyses of RCTs on therapies that improve NAFLD

Exposure	Author, year	Measure	Studies (n)	Subjects (n)	Cases (n)		Random effect model Effect size (95% CI)	***p***-value	I^**2**^ (%)	Heterogeneity ***p***-value	Small-study effects ***p***-value
Caffeine [26]	Shen2016	Liver fibrosis	2	NR	292	MD	−91.35 (−139.42, −43.27)	0.0002	0	0.74	NA
Green tea [25]	Ghanaei2018	ALT	4	234	122	MD	−12.81 (−18.17, −7.45)	<0.00001	9	0.35	0.75
Green tea [25]	Ghanaei2018	AST	4	234	122	MD	−10.91 (−19.66, −2.17)	0.01	80	0.002	0.32
Green tea [25]	Ghanaei2018	TG	3	163	87	MD	−31.86 (−40.62, −23.12)	<0.00001	0	0.53	0.71
Green tea [25]	Ghanaei2018	TC	3	163	87	MD	−27.57 (−36.17, −18.98)	<0.00001	3	0.36	0.82
Green tea [25]	Ghanaei2018	LDL	3	163	87	MD	−14.15 (−23.69, −4.60)	0.004	34	0.22	0.77
Green tea [25]	Ghanaei2018	BMI	4	234	122	MD	−2.08 (−2.81, −1.36)	<0.00001	0	0.49	0.06
Low carbohydrate diet [27]	Haghighatdoos2016	IHLC	4	NA	238	Mean percentage	−11.53% (−18.10, −4.96)	0.00085	83.2	<0.001	0.34
Omega-3 PUFAs [63]	Yan2018	ALT	14	937	NA	SMD	−0.50 (−0.88, −0.11)	0.000	86.4	<0.001	0.695
Omega-3 PUFAs [63]	Yan2018	AST	12	903	NA	SMD	−0.54 (−1.04, −0.05)	0.000	91.2	<0.001	0.733
Omega-3 PUFAs [63]	Yan2018	GGT	8	1121	NA	SMD	−0.48 (−0.64, −0.31)	0.013	41.6	0.101	0.945
Omega-3 PUFAs [63]	Yan2018	HOMR-IR	8	502	NA	SMD	−0.40 (−0.58, −0.22)	0.001	16.6	0.299	0.259
Omega-3 PUFAs [63]	Yan2018	Glucose	8	474	NA	SMD	−0.25 (−0.43, −0.06)	0.002	43	0.092	0.274
Omega-3 PUFAs [63]	Yan2018	TG	16	1075	NA	SMD	−0.47 (−0.76, −0.19)	0.002	79.6	<0.001	0.469
Omega-3 PUFAs [59]	Musa-Veloso 2017	Liver fat content	5	NA	NA	MD	−5.19% (−9.58, −0.97)	0.021	NA	NA	NA
Omega-3 PUFAs [59]	Musa-Veloso 2017	Grade of steatosis	7	NA	NA	MD	−0.71 (−0.99, −0.42)	<0.001	NA	NA	NA
Omega-3 PUFAs [28]	Parker2012	Liver fat	7	NA	355	ES	−0.97 (−0.58, −1.35)	<0.001	66.12	0.007	NA
Omega-3 PUFAs [58]	Yu2017	LDL	6	468	235	MD	−9.18 (−14.89, −3.47)	0.002	43	0.13	NA
Omega-3 PUFAs [58]	Yu2017	HDL	7	509	254	MD	4.81 (1.59, 8.03)	0.03	65	0.009	NA
Total exercise [60]	Smart 2016	Intrahepatic fat	21	1530	NA	SMD	−1.77 (−3.11, −0.42)	0.01	77	NA	0.1
Total exercise (irrespectively of weight change) [61]	Katsagoni2016	IHTG	10	540	325	SMD	−0.98 (−1.30, −0.66)	<0.001	62.1	0.002	0.012
Total exercise (irrespectively of weight change) [61]	Katsagoni2016	ALT	11	495	301	SMD	−0.39 (−0.66, −0.11)	0.006	55.3	0.008	0.015
Total exercise (irrespectively of weight change) [61]	Katsagoni2016	AST	9	494	373	SMD	−0.37 (−0.65, −0.09)	0.009	53.7	0.017	0.016
Total exercise (irrespectively of weight change) [61]	Katsagoni2016	WC	NA	564	NA	SMD	−0.6 (−0.78, −0.42)	<0.001	0	0.71	NA
Total exercise (irrespectively of weight change) [61]	Katsagoni2016	HOMA-IR	NA	564	NA	SMD	−0.76 (−1.47, −0.05)	<0.001	8	<0.001	NA
Total exercise (irrespectively of weight change) [22]	Keating2012	Liver fat	6	156	93	ES	−0.37 (−0.69, −0.06)	0.02	NA	NA	NA
Exercise (AEx) [ 61]	Katsagoni2016	IHTG	5	119	68	SMD	−0.84 (−1.27, −0.42)	<0.001	66.6	NA	NA
Exercise (RT) [61]	Katsagoni2016	IHTG	3	133	72	SMD	−1.05 (−1.87, −0.24)	0.011	65.1	NA	NA
Exercise (AEx + RT) [ 61]	Katsagoni2016	IHTG	3	61	36	SMD	−1.54 (−2.56, −0.52)	0.003	60.5	NA	NA
Exercise (continuous MIT) [61]	Katsagoni2016	IHTG	2	229	93	SMD	−0.86 (−1.36, −0.34)	0.001	63.5	NA	NA
Exercise (low-to-moderate volume MIT) [61]	Katsagoni2016	IHTG	4	234	124	SMD	−0.50 (−0.77, −0.23)	<0.001	0	NA	NA
Exercise (AEx) [62]	Zou2018	ALT	20	846	134	WMD	−17.04 (−38.08,-4.00)	0.01	0	NA	0.04
Exercise (RT) [62]	Zou2018	ALT	20	846	71	WMD	−17.33 (− 43.90, −8.22)	<0.001	7.6	NA	0.59
Exercise (AEx + RT) [62]	Zou2018	ALT	20	846	26	WMD	−32.12 (− 66.11, −1.87)	<0.001	NA	NA	NA
Exercise (AEx) [62]	Zou2018	AST	17	790	110	WMD	−5.83 (−12.21, −0.45)	<0.001	61.6	NA	0.03
Exercise (RT) [62]	Zou2018	AST	17	790	60	WMD	−4.38 (−20.58, 11.83)	<0.001	0	NA	NA
Exercise (AEx) [62]	Zou2018	HOMR-IR	11	492	69	WMD	−0.17 (−0.69, 0.36)	<0.001	0	NA	0.02
Exercise (RT) [62]	Zou2018	HOMR-IR	11	492	11	WMD	−1.70 (− 5.61, 2.21)	<0.001	NA	NA	NA
Exercise (AEx + RT) [62]	Zou2018	HOMR-IR	11	492	26	WMD	−0.52 (−1.51, 0.41)	<0.001	NA	NA	NA
Exercise (AEx) [62]	Zou2018	BMI	20	13,426	846	WMD	−1.55 (− 3.52, −0.42)	<0.001	59.4	NA	0.19
Exercise (RT) [62]	Zou2018	BMI	20	846	71	WMD	−1.81 (−3.80, −0.18)	<0.001	0	NA	0.07
Exercise (AEx + RT) [62]	Zou2018	BMI	20	846	26	WMD	−2.09 (−4.07, −0.10)	<0.001	NA	NA	NA
Weight loss [29]	Koutoukidis 2019	ALT	21	2558	1496	MD	−9.18 (−13.12, −6.50)	<0.001	97	<0.001	NA
Weight loss [29]	Koutoukidis 2019	AST	19	2558	1446	MD	−4.84 (−7.13, −2.38)	0.0001	96	<0.00001	NA
Weight loss [29]	Koutoukidis 2019	GGT	9	1774	1124	MD	−4.35 (−7.67, −1.04)	0.01	96	<0.00001	NA
Weight loss [29]	Koutoukidis 2019	Liver stiffness	4	271	151	SMD	−1.11 (−1.91, −0.32)	0.006	94	<0.00001	NA
Weight loss [29]	Koutoukidis 2019	Liver steatosis	11	765	405	SMD	−1.48 (−2.27, −0.7)	<0.001	94	<0.01	NA
Weight loss [29]	Koutoukidis 2019	NAS	5	164	93	SMD	−0.92 (−1.75, −0.09)	0.03	95	<0.001	NA

*NAFLD* Nonalcoholic fatty liver disease, *IHCL* Intrahepatocellular lipids, *WHR* Waist-tohip ratio, *WC* Waist circumference, *BMI* Body mass index, *Omega-3 PUFAs* Omega-3 Polyunsaturated fatty acids, *NASH* Non-alcoholic steatohepatitis, *NAS* Nonalcoholic activity score, *ALT* Alanine aminotransferase, *AST* Aspartate aminotransferase, *TG* Triglyceride, *TC* Total cholesterol, *LDL-C* Low-density lipoprotein cholesterol, *BMI* Body mass index, *IHLC* Intrahepatic lipid content, *GGT* G-glutamyl transferase, *HOMA-IR* Homeostasis model assessment of insulin resistance, *HDL-C* High density lipoprotein, *AEx* Aerobic exercise training, *RT* Resistance training, *MIT* Moderate-intensity, *HIT* High-intensity training, *HIIT* High-intensity interval training, *IHTG* Intrahepatic triglyceride, *MIT* Moderate inten-sity, *ALP* Alkaline phosphatase, *MD* Mean difference, *SMD* Standardized mean difference, *WMD* Weighted mean difference, *ES* Effect size, *OR* Odds ratio, *RR* Relative risk, *CI* Confidence interval, *NA* Not available

##### Green tea

Green tea consumption not only reduced the risk of NAFLD, but also seemed to have efficacy in NAFLD treatment. It resulted in a significant reduction of ALT (MD, −12.81 U/L; −18.17, −7.45) and AST (MD, −10.91 U/L; −19.66, −2.17); decreased plasma concentrations of TG (MD,-31.86 mg/dl; −40.62, −23.12), TC (MD, −27.57 mg/dl; −36.17, −18.98), and LDL (MD, −14.15 mg/dl; −23.69, −4.60); and decreased BMI (MD, −2.08 kg/m^2^; −2.81, −1.36) [25] (Table 1).

##### Low carbohydrate diet

Low carbohydrate diet decreased intrahepatic lipid content (IHLC) (MD, −11.53%; −18.10, −4.96), but did not significantly affect the concentration of liver enzymes in patients with NAFLD [27] (Table 1).

##### Omega-3 polyunsaturated fatty acids supplementation

Compared with placebo-treated participants, omega-3 polyunsaturated fatty acids (omega-3 PUFAs) intake could improve ALT (SMD, −0.50; −0.88, −0.11), AST (SMD, −0.54; −1.04, −0.05), GGT (SMD, −0.48; −0.64, −0.31), HOMA-IR (SMD, −0.40; −0.58, −0.22), glucose (SMD, −0.25; −0.43, −0.06), and TG (SMD,-0.47; −0.76, −0.19) in patients with NAFLD [62]. Omega-3 PUFAs supplementation significantly reduced liver fat content (MD, −5.19%; −9.58, −0.97) [58], and grade of steatosis (MD, −0.71; −0.99, −0.42) [58]. There was a significant pooled effect size (ES) for the efficacy of omega-3 PUFAs therapy on liver fat (ES, −0.97;-0.58, −1.35) [28]. The treatment of omega-3 PUFAs decreased LDL (MD, −9.18; −14.89, −3.47) and increased HDL (MD, 4.81; 1.59, 8.03) in NAFLD patients [57] (Table 1).

##### Exercise

All interventions for NAFLD patients were categorized by exercise type, intensity, and volume including total exercise, total exercise (irrespective of weight change), total exercise (no significant weight loss), aerobic exercise training (AEx), resistance training (RT), AEx plus RT, continuous moderate-intensity training (MIT), continuous high-intensity training (HIT), continuous high-intensity interval training (HIIT), low-to-moderate volume MIT, moderate-to-high volume MIT. Compared to usual care, total exercise had a positive effect on intrahepatic fat (SMD, −1.77; −3.11, −0.42) [59]; total exercise (irrespectively of weight change) reduced IHTG (SMD,-0.98; −1.30, −0.66), ALT (SMD, −0.39; −0.66, −0.11), AST (SMD, −0.37; −0.65, −0.09), WC (SMD, −0.60; −0.78, −0.42), HOMA-IR (SMD, −0.76; −1.47, −0.05), and liver fat (ES, −0.37;-0.69, −0.06) [22, 60].

Subgroup analyses revealed that AEx, RT, AEx plus RT, continuous MIT, and low-to-moderate volume MIT all improved IHTG ((SMD, −0.84; −1.27, −0.42), (SMD, −1.05; −1.87, −0.24), (SMD, −1.54; −2.56, −0.52), (SMD, −0.86; −1.36, −0.34), and (SMD, −0.50; −0.77, −0.23), respectively) [60]. Moreover, AEx, RT, and AEx plus RT all improved ALT ((WMD, −17.04;−38.08, −4.00), (WMD, −17.33; − 43.90, −8.22), and (WMD, −32.12; − 66.11, −1.87), respectively); AEx and RT improved AST ((WMD, −5.83; −12.21, −0.45) and (WMD, −4.38; −20.58, 11.83), respectively); AEx, RT, and AEx plus RT all improved HOMR-IR ((WMD, −0.17; −0.69, 0.36), (WMD, −1.70; −5.61, 2.21), and (WMD, −0.52; −1.51, 0.41), respectively); AEx, RT, and AEx plus RT all improved BMI ((WMD, −1.55; − 3.52, −0.42), (WMD, −1.81; −3.80, −0.18), and (WMD, −2.09; −4.07, −0.10), respectively)[62] (Table 1).

##### Weight loss

In patients with NAFLD, compared with no or minimal or lower-intensity interventions, more-intensive weight loss interventions (−3.61 kg; −5.11, −2.12) improved blood biomarkers (ALT (MD, −9.81; −13.12, −6.50), AST (MD, −4.84; −7.31, −2.38), and GGT (MD, −4.35; −7.67, −1.04)) as well as radiologic and histologic markers of liver stiffness (SMD, −1.11; −1.91, −0.32), liver steatosis (SMD, −1.48; −2.27, −0.70), and NAS (MD, −0.92; −1.75, −0.09) [29] (Table 1).

#### Therapies that do not significantly improve NAFLD

Omega-3 PUFAs supplementation did not significantly improve TC in patients with NAFLD [62]. Total exercise (irrespectively of weight change), AEx, RT, and AEx plus RT did not significantly improve serum liver enzyme (GGT), serum liver enzymes (ALT, AST, and GGT), serum liver enzymes (ALT, AST, and GGT), and serum liver enzymes (ALT, AST, and GGT), respectively) [60]. In addition, weight loss did not improve ALP and the histologic scores for inflammation, ballooning, or fibrosis in NAFLD patients [29] (Supplementary Table 6).

#### Strength of epidemiologic evidence

The grading of evidence from the meta-analyses of observational studies was presented.

in (Table 2). Sugar-sweetened soda increased the risk of NAFLD with a high epidemiologic evidence. 7 risk factors (soft drinks, hypercaloric fructose diet (IHLC), obesity, central obesity (Per 1-unit increase in WC), central obesity (Per 1-unit increase in BMI), hyperuricemia, and hyperuricemia (NAS)) and 3 protective factors (modest alcohol (less than 40 g/day), modest alcohol (less than 40 g/day) (NASH), and coffee) showed moderate epidemiologic evidence with respect to NAFLD. 7 risk factors (smoking, passive smoking, former smoking, sugar sweetened beverages (SSB), red meat, short sleep, and central obesity (WHR)) and 4 protective factors (coffee (liver fibrosis), green tea (liver steatosis), nuts, and weight loss (NASH)) showed low epidemiologic evidence in relation to NAFLD.

**Table 2:** The strength of epidemiologic evidence of 22 meta-analyses of observational studies that affect the risk of NAFLD

Exposure	Measure	Reference	Precision of the estimate		Consistency of results	No evidence of small-study effects	*Grade*
			>1000 disease cases	***P*** < 0.001	I^**2**^ < 50% and Cochran Q test ***P*** > .10	***P*** > 0.1	
**15 factors that increased the risk of NAFLD**							
Smoking	NAFLD	Rezayat2017	Yes	No	Yes	Yes	Low
Passive smoking	NAFLD	Rezayat2017	No	No	No	Yes	Low
Former smoking	NAFLD	Rezayat2017	No	No	Yes	Yes	Low
Soft drinks	NAFLD	He2020	Yes	Yes	Yes	No	Moderate
sugar sweetened beverages	NAFLD	Asgar-Taee2018	Yes	No	Yes	Yes	Low
Sugar-Sweetened Soda	NAFLD	Wijarnpreecha2015	Yes	Yes	Yes	Yes	High
Hypercaloric fructose diet	IHLC	Chung2014	No	Yes	Yes	Yes	Moderate
Red meat	NAFLD	He2020	Yes	No	No	No	Low
Short sleep	NAFLD	Wijarnpreecha2016	Yes	No	Yes	Yes	Low
Obesity	NAFLD	Li2016	Yes	Yes	No	Yes	Moderate
Per 1-unit increase in WC	NAFLD	Pang2015	Yes	Yes	No	Yes	Moderate
Per 1-unit increase in BMI	NAFLD	Pang2015	Yes	Yes	No	Yes	Moderate
WHR	NAFLD	Pang2015	No	No	No	Yes	Low
Hyperuricemia	NAFLD	Darmawan2017	Yes	Yes	No	Yes	Moderate
Hyperuricemia	NAS	Jaruvongvanich2017	No	Yes	Yes	Yes	Moderate
**7 factors that decreased the risk of NAFLD**							
Modest alcohol	NAFLD	Sookoian2014	Yes	Yes	No	Yes	Moderate
Modest alcohol	NASH	Sookoian2014	No	Yes	Yes	Yes	Moderate
Coffee	NAFLD	Chen2018	Yes	Yes	No	Yes	Moderate
Coffee	Liver fibrosis	Wijarnpreecha2017	No	Yes	No	No	Low
Green tea	Liver steatosis	Yin2015	No	No	No	Yes	Low
Nut	NAFLD	He2020	Yes	No	Yes	Yes	Low
Weight loss	NASH	Koutoukidis2019	No	No	Yes	No	Low

*WHR* Waist-tohip ratio, *NAFLD* Nonalcoholic fatty liver disease, *HCL* Intrahepatocellular lipids, Omega-3 PUFAs, *WC* Waist-tohip ratio, *BMI* Body mass index, *NAS* Non-alcoholic activity score, *NASH* Non-alcoholic steatohepatitis; omega-3 polyunsaturated fatty acids

NOTE. The strength of epidemiologic evidence was rated as follows:

High, if all criteria were satisfied: precision of the estimate (*P* < .001 and > 1000 disease cases), consistency of results (I^2^ < 50% and Cochran Q test *P* > .10), and no evidence of smallstudy effects (*P* > .10)

Moderate, if a maximum of 1 criterion was not satisfied and a *P* < .001 was found

Low, in other cases (*P* < .05)

The other 15 putative factors did not show statistically significant associations with respect to NAFLD risk (Supplementary Table 7). In these factors, 26.7% (4/15) meta-analyses showed no large heterogeneity (I^2^ < 50%) and 73.3% (11/15) had a large heterogeneity (I^2^ ≥50%). Moreover, 73.3% (11/15) meta-analyses showed no small study-effects (*P* > 0.1).

Evidence from the meta-analyses of RCTs was presented in (Table 1). In the therapies that improve NAFLD, 79.2% (38/48) treatment interventions had nominally significant summary results at *P* < 0.01 and 20.8% (10/48) at 0.01 ≤ *P* < 0.05. In these treatment interventions, 37.5% (18/48) showed no large heterogeneity (I^2^ < 50%), 47.9% (23/48) had a large heterogeneity (I^2^ ≥50%), and 14.6% (7/48) were not available on heterogeneity due to lack of the concerning data in original meta-analyses. Furthermore, 29.2% (14/48) showed no small study effects (*P* > 0.1), 18.8%(9/48) had small study effects (*P* ≤0.1), and 52.0% (25/48) were not available on small study effects. Only 7 treatment interventions (14.6%) reported a *P* < 0.01 and had no evidence of large heterogeneity and small study effects (green tea (ALT), green tea (TG), green tea (TC), green tea (LDL), omega-3 PUFAs (HOMR-IR), omega-3 PUFAs (glucose), and exercise (RT) (ALT)). In the treatment interventions with improvement of liver fat content or hepatic histopathology, caffeine (liver fibrosis), low carbohydrate diet (IHLC), omega-3 PUFAs (liver fat), total exercise (irrespectively of weight change) (IHTG), exercise (AEx) (IHTG), exercise (AEx + RT) (IHTG), exercise (continuous MIT) (IHTG), exercise (low-to-moderate volume MIT) (IHTG), weight loss (liver stiffness), and weight loss (liver steatosis) interventions showed a *P* < 0.01, but had a large heterogeneity (I^2^ ≥50%) and/or small study effects (*P* ≤0.1) (or were not available), whereas omega-3 PUFAs (liver fat content), total exercise (intrahepatic fat), total exercise (irrespectively of weight change) (liver fat), exercise (RT) (IHTG), weight loss (NAS) interventions showed the lowest strength of evidence (had a0.01 ≤ *P* < 0.05 and a large heterogeneity (I^2^ ≥50%) and/or small study effects (*P* ≤0.1) and/or were not available).

The other 15 treatment interventions did not show statistically significant associations in relation to NAFLD (*P*  ≥0.05) (Supplementary Table 6). In these treatment interventions, 26.7% (4/15) showed no large heterogeneity (I^2^ < 50%), 33.3% (5/15) had a large heterogeneity (I^2^ ≥50%), and 40.0% (6/15) were not available on heterogeneity due to lack of the concerning data in original meta-analyses. On the other hand, 13.3% (2/15) treatment interventions had small study effects (*P*  0.1) and 86.7% (13/15) were not available with respect to small study-effects due to lack of the concerning data in original meta-analyses.

## Discussion

### Main findings

The influence of lifestyle as well as metabolic syndrome on NAFLD incidence or treatment has been examined in many published meta-analyses. This umbrella review provided a comprehensive overview of reported associations between lifestyle or metabolic syndrome and the risk or treatment of NAFLD by incorporating evidence from meta-analyses of observational studies and RCTs. We also further evaluated the methodological quality of the meta-analyses and quality of evidence for all these associations by following criteria that have been previously applied to appraise the strength of epidemiologic evidence in several research publications [32, 37].

We included 35 publications, Which comprised 100 meta-analyses (37 meta-.

analyses of observational studies and 63 meta-analyses of RCTs). The methodological quality was high for 97% of the published meta-analyses. For the meta-analyses of observational studies, the quality of evidence was graded as high only for sugar-sweetened soda, which increased the risk of NAFLD; The quality of evidence was graded as moderate for 2 dietary factors (soft drinks, hypercaloric fructose diet (IHLC)), 3 obesity factors (obesity, central obesity (Per 1-unit increase in WC), and central obesity (Per 1-unit increase in BMI)), and 2 metabolic factors (hyperuricemia and hyperuricemia (NASH)) that increased the risk of NAFLD, and for 3 dietary factors (modest alcohol (less than 40 g/day), modest alcohol (less than 40 g/day) (NASH), and coffee) that decreased incidence of NAFLD; For the other associations (another 7 risk and 4 protective factors with respect to NAFLD), the quality of evidence was low and further investigation is needed.

For evidence from the meta-analyses of RCTs, although 79.2% (38/48) treatment interventions had *P* < 0.01 in the meta-analyses of nominally significant summary results (*P* < 0.05), only 7 treatment interventions (4 green tea interventions, 2 omega-3 PUFAs interventions, and 1 exercise (RT) intervention) had a *P* < 0.01, with no evidence of large heterogeneity and small study effects. These therapies were only associated with an improvement of liver enzymes, blood lipids and blood glucose rather than histological changes of liver. In the therapies that improved liver fat content or hepatic histopathology, 3 dietary interventions (caffeine, low carbohydrate diet, and omega-3 PUFAs), 5 exercise interventions (total exercise (irrespectively of weight change), exercise (AEx), exercise (AEx + RT), exercise (continuous MIT), and exercise (low-to-moderate volume MIT)), and 2 weight loss interventions (weight loss (liver stiffness), and weight loss (liver steatosis)) achieved *P* < 0.01, but large heterogeneity and/or evidence of bias existed in these meta-analyses, indicating that these associations should be interpreted with caution.

### Comparison with other studies and possible explanations

Existing guidelines hold components of metabolic syndrome (obesity, T2DM, hypertension, dyslipidemia) and intake of sugar-sweetened beverages as risk factors associated with NAFLD [23, 65, 66]. Moreover, the umbrella review by Neuenschwander et al. [67] showed that sugar sweetened beverages increased T2DM incidence with a high quality of evidence. This information correlates with our results that sugar-sweetened soda, soft drinks, obesity, central obesity (Per 1-unit increase in WC), and central obesity (Per 1-unit increase in BMI) were associated with an increased incidence of NAFLD, for which we found high/moderate quality of evidence. Sugar sweetened beverages are not only a major risk factor for weight gain and obesity [68], but also have a high glycaemic index [69], which may contribute to the risk of NAFLD. Fructose is a source of excess calories, and a high fructose intake is associated with NAFLD [70]. Fructose increases hepatic de novo lipogenesis in a dose-dependent fashion [71] and de novo lipogenesis has been shown to be abnormally unregulated inpatients with NAFLD [72]. Artificial sweeteners or sugar substitutes are food additives that provide a sweet taste and are also known as low-calorie or non-calorie sweeteners. It has a potential role in microbiota alteration and dysbiosis [73]. Our result showed that hypercaloric fructose diet increased intrahepatic lipid content in healthy male adults with moderate quality of evidence, which was consistent with the aforementioned results. Moreover, we found that hyperuricemia was associated with an increased risk of NAFLD and NASH with moderate quality of evidence. Similarly, the umbrella review by Li et al. indicated that hyperuricemia increased the risk of T2DM and metabolic syndrome [74]. The mechanistic role of uric acid in NAFLD is potentially involved in multiple biological processes, including stimulating inflammation, inducing oxidative stress, and amplifying the lipogenic effects of fructose [46, 75, 76]. Many aspects of childhood or adolescent and adult NAFLD were considered inconsistent, including prevalence, histology, diagnosis and management [77]. Studies that included children were excluded from our analysis.

Alcohol consumption up to 30 g/day (men) or 20 g /day (women) is insufficient to induce alcoholic steatosis and might even be protective against NAFLD, NASH and fibrosis as compared with total abstinence [65]. One guideline states that moderate consumption of alcohol reduces incidence of T2DM [78]. The umbrella review by Neuenschwander et al. [67] indicated that there was an inverse association between moderate total alcohol consumption (12–24 g/day) or coffee intake and incidence of T2DM, with high or moderate quality of evidence, respectively. In addition, Poole et al. reported that coffee consumption was associated with a decreased risk of NAFLD, liver fibrosis, and liver cirrhosis in an umbrella review [79]. Our results indicated a beneficial association of NAFLD incidence with intake of modest alcohol (less than 40 g/day), modest alcohol (less than 40 g/day) (NASH), and coffee with moderate quality of evidence, which supports the aforementioned findings. Regarding the mechanisms, several observational studies indicated that light or moderate alcohol consumption increases insulin sensitivity [80–82]. However, as alcohol causes adverse health effects such as liver cirrhosis, and increased risk for cancers [83], translation of these results into recommendations have to be considered carefully. The potential mechanisms for the hepatoprotection of coffee involve caffeine, phenolic compounds, and melanoidins. Caffeine has been implicated in increasing insulin sensitivity [84] and restraining the hepatic fibrinogenesis pathway by downregulating the production of connective tissue growth factor induced by transforming growth factor-β1, by upregulating the peroxisome–proliferator-activatedreceptor γ (PPARγ), and by inhibiting the synthesis of focal adhesion kinase and actin [85]. Phenolic compounds, melanoidins, and caffeine are responsible for antioxidant effects that prevent free radical tissue damage by reducing reactive oxygen species, which, in turn, play a central part in the inflammation processes of NAFLD [86].

The umbrella reviews by Yi et al. [87]. and Neuenschwander et al. [67]. indicated that tea consumption was associated with a reduced risk of T2DM; also, Yi et al. [87]. showed that high consumption of green tea was associated with a reduced risk of liver cancer. Current guidelines indicate that omega-3 PUFAs can improve blood lipid profile and reduce liver fat [23, 88, 89]. Grosso et al. reported that incremental intake of caffeine significantly decreased the risk of T2DM [90]. Lifestyle modifications consisting of energy restriction, exercise, and weight loss are recommended as the first-line treatment for patients with NAFLD by guidelines, and these treatment interventions alone or their conjunction can improve liver biochemistry, steatosis, even fibrosis [23, 65, 66, 91, 92]. Our results indicated that green tea, omega-3 PUFAs, and exercise (RT) effectively improve liver enzymes, blood lipids and blood glucose rather than histological changes of the liver, with the higher strength of epidemiologic evidence (had a *P* < 0.01 and had no evidence of large heterogeneity and small study effects); but some other treatment interventions (caffeine, low carbohydrate diet, omega-3 PUFAs, exercise (different exercise type, intensity, or volume), and weight loss, which can improve liver fat content or hepatic histopathology, had lower strength of epidemiologic evidence (had a *P* < 0.01 but had a large heterogeneity and/or small study effects). Therefore, multi-center, prospective, large sample RCTs are needed to further investigate the therapeutic effect of these lifestyle modifications, especially exercise and weight loss on liver fat content, NASH, and liver fibrosis. Our results support the aforementioned findings and guideline recommendations.

The mechanisms by which the above dietary ingredients are responsible for therapeutic effects of NAFLD involve many factors. Experimental evidence from in vitro systems and animal models supports a role of green tea or its catechins in protecting against NAFLD by decreasing intestinal lipid and carbohydrate absorption, by decreasing adipose lipolysis and hepatic de novo lipogenesis, by stimulating hepatic β-oxidation and thermogenesis, and by improving insulin sensitivity [93]. Furthermore, green tea displays the hepatoprotective effects through its antioxidant and anti-inflammatory properties [94, 95]. Omega-3 PUFAs influence NAFLD through several mechanisms. They has been shown to downregulate sterol-regulatory-element-binding protein 1c (SREBP-1c) and upregulate peroxisome–proliferator-activated receptor α (PPARα), which would favor fatty acid oxidation and reduce steatosis [96]. Moreover, Omega-3 PUFAs can give rise to resolvins, which are anti-inflammatory [97]. A possible explanation for beneficial effects of low carbohydrate diets in patients with NAFLD may be related to enhanced lipid oxidation that is induced by energy and carbohydrate restriction [98, 99].

### Strengths and limitations

In this umbrella review, we systematically and comprehensively presented the evidence of the associations between lifestyle or metabolic syndrome and NAFLD incidence or treatment by incorporating information from meta-analyses of observational studies and RCTs. We also evaluated the methodological quality and quality of evidence by using validated tools [31–37]. Furthermore, we analyzed the extent of heterogeneity and publication bias.

This umbrella review had several limitations. Firstly, our umbrella review focused on existing meta-analyses and therefore outcomes that were not assessed in any published meta-analyses are not included in the review. Secondly, even though the total number of included studies was large, for some associationsthe number of studies included in the meta-analysis was small, which might cause publication bias. Thirdly, we did not evaluate the quality of the individual studies, since this should be the responsibility of the authors of the original meta-analysis and it was beyond the scope of the current umbrella review. Finally, we did not perform subgroup analysis (eg, by sex or geographical locations) or sensitivity analysis (eg, exclusion of studies at high risk of bias).

## Conclusions

Although the associations of lifestyle as well as metabolic syndrome with the risk or treatment of NAFLD have been examined in a large number of published meta-analyses, the quality of evidence was only high for the association of sugar-sweetened soda with increased NAFLD risk, and only 7 treatment interventions (4 green tea interventions, 2 omega-3 PUFAs interventions, and 1 exercise (RT) intervention) had the higher strength of epidemiologic evidence, demonstrating improvement of liver enzymes, blood lipids and blood glucose rather than histological changes of liver. Robust clinical RCTs are needed to further investigate the associations between lifestyle modifications and incidence of or the therapeutic effects on NAFLD.

## Supplementary Information

**Additional file 1.**

**Additional file 2.**

**Additional file 3.**

**Additional file 4.**

**Additional file 5.**

**Additional file 6.**

**Additional file 7.**

## Data Availability

The datasets analysed in the current study are available from the corresponding author on reasonable request.

## Declarations

### Competing of interests

The authors declared there is no confict of interest.

## References

[1] Rinella ME (2015). Nonalcoholic fatty liver disease: a systematic review. JAMA.

[2] Diehl AM, Day C (2017). Cause, pathogenesis, and treatment of nonalcoholic steatohepatitis. N Engl J Med.

[3] Younossi ZM, Koenig AB, Abdelatif D, Fazel Y, Henry L, Wymer M (2016). Global epidemiology of nonalcoholic fatty liver disease-meta-analytic assessment of prevalence, incidence, and outcomes. Hepatology.

[4] Clark JM, Brancati FL, Diehl AM (2003). The prevalence and etiology of elevated aminotransferase levels in the United States. Am J Gastroenterol.

[5] Nd AM (2019). Non-alcoholic fatty liver disease, an overview. Integr Med (Encinitas).

[6] Rhee EJ (2019). Nonalcoholic fatty liver disease and diabetes: an epidemiological perspective. Endocrinol Metab (Seoul).

[7] Lindenmeyer CC, McCullough AJ (2018). The natural history of nonalcoholic fatty liver disease-an evolving view. Clin Liver Dis.

[8] Zelber-Sagi S, Lotan R, Shlomai A (2012). Predictors for incidence and remission of NAFLD in the general population during a seven-year prospective follow-up. J Hepatol.

[9] Kasturiratne A, Weerasinghe S, Dassanayake AS (2013). Influence of non-alcoholic fatty liver disease on the development of diabetes mellitus. J Gastroenterol Hepatol.

[10] Miele L, Targher G (2015). Understanding the association between developing a fatty liver and subsequent cardio-metabolic complications. Expert Rev Gastroenterol Hepatol.

[11] Chitturi S, Wong VW, Farrell G (2011). Nonalcoholic fatty liver in Asia: firmly entrenched and rapidly gaining ground. J Gastroenterol Hepatol.

[12] Vernon G, Baranova A, Younossi ZM (2011). Systematic review: the epidemiology and natural history of non-alcoholic fatty liver disease and non-alcoholic steatohepatitis in adults. Aliment Pharmacol Ther.

[13] Huang TD, Behary J, Zekry A (2020). Non-alcoholic fatty liver disease (NAFLD): a review of epidemiology, risk factors, diagnosis and management. Intern Med J..

[14] Ahn J, Jun DW, Lee HY, Moon JH (2019). Critical appraisal for low-carbohydrate diet in nonalcoholic fatty liver disease: review and meta-analyses. Clin Nutr.

[15] Wijarnpreecha K, Thongprayoon C, Panjawatanan P, Ungprasert P (2016). Short sleep duration and risk of nonalcoholic fatty liver disease: a systematic review and meta-analysis. J Gastroenterol Hepatol.

[16] Akhavan Rezayat A, Dadgar Moghadam M, Ghasemi Nour M (2018). Association between smoking and non-alcoholic fatty liver disease: a systematic review and meta-analysis. SAGE Open Med.

[17] Yuan H, Yu C, Li X (2015). Serum uric acid levels and risk of metabolic syndrome: a dose-response meta-analysis of prospective Studies. J Clin Endocrinol Metab.

[18] Dai W, Ye L, Liu A (2017). Prevalence of nonalcoholic fatty liver disease in patients with type 2 diabetes mellitus: a meta-analysis. Medicine (Baltimore).

[19] Chen YP, Lu FB, Hu YB, Xu LM, Zheng MH, Hu ED (2019). A systematic review and a dose-response meta-analysis of coffee dose and nonalcoholic fatty liver disease. Clin Nutr.

[20] Marventano S, Salomone F, Godos J (2016). Coffee and tea consumption in relation with non-alcoholic fatty liver and metabolic syndrome: a systematic review and meta-analysis of observational studies. Clin Nutr.

[21] Sookoian S, Castaño G (2014). Modest alcohol consumption decreases the risk of non-alcoholic fatty liver disease: a meta-analysis of 43 175 individuals. Gut..

[22] Keating SE, Hackett DA, George J, Johnson NA (2012). Exercise and non-alcoholic fatty liver disease: a systematic review and meta-analysis. J Hepatol.

[23] Chalasani N, Younossi Z, Lavine JE (2018). The diagnosis and management of nonalcoholic fatty liver disease: practice guidance from the American Association for the study of liver diseases. Hepatology.

[24] Osterberg L, Blaschke T (2005). Adherence to medication. N Engl J Med.

[25] Mansour-Ghanaei F, Hadi A, Pourmasoumi M, Joukar F, Golpour S, Najafgholizadeh A (2018). Green tea as a safe alternative approach for nonalcoholic fatty liver treatment: a systematic review and meta-analysis of clinical trials. Phytother Res.

[26] Shen H, Rodriguez AC, Shiani A (2016). Association between caffeine consumption and nonalcoholic fatty liver disease: a systemic review and meta-analysis. Ther Adv Gastroenterol.

[27] Haghighatdoost F, Salehi-Abargouei A, Surkan PJ, Azadbakht L (2016). The effects of low carbohydrate diets on liver function tests in nonalcoholic fatty liver disease: a systematic review and meta-analysis of clinical trials. J Res Med Sci.

[28] Parker HM, Johnson NA, Burdon CA, Cohn JS, O'Connor HT, George J (2012). Omega-3 supplementation and non-alcoholic fatty liver disease: a systematic review and meta-analysis. J Hepatol.

[29] Koutoukidis DA, Astbury NM, Tudor KE (2019). Association of weight loss interventions with changes in biomarkers of nonalcoholic fatty liver disease: a systematic review and meta-analysis. JAMA Intern Med.

[30] Moher D, Liberati A, Tetzlaff J, Altman DG, PRISMA Group (2009). Preferred reporting items for systematic reviews and meta-analyses: the PRISMA statement. BMJ.

[31] Shea BJ, Grimshaw JM, Wells GA (2007). Development of AMSTAR: a measurement tool to assess the methodological quality of systematic reviews. BMC Med Res Methodol.

[32] Piovani D, Danese S, Peyrin-Biroulet L, Nikolopoulos GK, Lytras T, Bonovas S (2019). Environmental risk factors for inflammatory bowel diseases: an umbrella review of meta-analyses. Gastroenterology..

[33] Theodoratou E, Tzoulaki I, Zgaga L, Ioannidis JP (2014). Vitamin D and multiple health outcomes: umbrella review of systematic reviews and meta-analyses of observational studies and randomised trials. BMJ.

[34] Tsilidis KK, Kasimis JC, Lopez DS, Ntzani EE, Ioannidis JP (2015). Type 2 diabetes and cancer: umbrella review of meta-analyses of observational studies. BMJ.

[35] Belbasis L, Bellou V, Evangelou E, Ioannidis JP, Tzoulaki I (2015). Environmental risk factors and multiple sclerosis: an umbrella review of systematic reviews and meta-analyses. Lancet Neurol.

[36] Johnson VE (2013). Revised standards for statistical evidence. Proc Natl Acad Sci U S A.

[37] Ioannidis JP, Tarone R, McLaughlin JK (2011). The false-positive to false-negative ratio in epidemiologic studies. Epidemiology.

[38] Altman DG, Bland JM (2011). How to obtain the *P* value from a confidence interval. BMJ.

[39] Egger M, Davey Smith G, Schneider M, Minder C (1997). Bias in meta-analysis detected by a simple, graphical test. BMJ.

[40] Asgari-Taee F, Zerafati-Shoae N, Dehghani M, Sadeghi M, Baradaran HR, Jazayeri S (2019). Association of sugar sweetened beverages consumption with non-alcoholic fatty liver disease: a systematic review and meta-analysis. Eur J Nutr.

[41] Wijarnpreecha K, Thongprayoon C, Edmonds PJ, Cheungpasitporn W (2016). Associations of sugar- and artificially sweetened soda with nonalcoholic fatty liver disease: a systematic review and meta-analysis. QJM.

[42] He K, Li Y, Guo X, Zhong L, Tang S (2020). Food groups and the likelihood of non-alcoholic fatty liver disease: a systematic review and meta-analysis. Br J Nutr..

[43] Chung MPHM, Lichtenstein AH (2014). Fructose, high fructose corn syrup, sucrose, and nonalcoholic fatty liver disease.

[44] Chiu S, Sievenpiper JL, De Souza RJ (2014). Effect of fructose on markers of non-alcoholic fatty liver disease (NAFLD): a systematic review and meta-analysis of controlled feeding trials. Eur J Clin Nutr.

[45] Li L, Liu DW, Yan HY, Wang ZY, Zhao SH, Wang B (2016). Obesity is an independent risk factor for non-alcoholic fatty liver disease: evidence from a meta-analysis of 21 cohort studies. Obes Rev.

[46] Darmawan G, Hamijoyo L, Hasan I (2017). Association between serum uric acid and non-alcoholic fatty liver disease: a meta-analysis. Acta Med Indones.

[47] Chen YP, Lu FB, Hu YB, Xu LM, Zheng MH, Hu ED (2019). A systematic review and a dose-response meta-analysis of coffee dose and nonalcoholic fatty liver disease. Clin Nutr..

[48] Wijarnpreecha K, Thongprayoon C, Ungprasert P (2017). Coffee consumption and risk of nonalcoholic fatty liver disease: a systematic review and meta-analysis. Eur J Gastroenterol Hepatol.

[49] Yin X, Yang J, Li T (2015). The effect of green tea intake on risk of liver disease: a meta analysis. Int J Clin Exp Med.

[50] Ahn J, Jun DW, Lee HY, Moon JH (2019). Critical appraisal for low-carbohydrate diet in nonalcoholic fatty liver disease: Review and meta-analyses. Clin Nutr..

[51] Wijarnpreecha K, Panjawatanan P, Lekuthai N, Thongprayoon C, Cheungpasitporn W, Ungprasert P (2017). Hyperuricaemia and risk of nonalcoholic fatty liver disease: a meta-analysis. Liver Int.

[52] Gong S, Song J, Wang L, Zhang S, Wang Y (2016). Hyperuricemia and risk of nonalcoholic fatty liver disease: a systematic review and meta-analysis. Eur J Gastroenterol Hepatol.

[53] Zhou Y, Wei F, Fan Y (2016). High serum uric acid and risk of nonalcoholic fatty liver disease: a systematic review and meta-analysis. Clin Biochem.

[54] Pang Q, Zhang JY, Song SD (2015). Central obesity and nonalcoholic fatty liver disease risk after adjusting for body mass index. World J Gastroenterol.

[55] Jaruvongvanich V, Ahuja W, Wirunsawanya K, Wijarnpreecha K, Ungprasert P (2017). Hyperuricemia is associated with nonalcoholic fatty liver disease activity score in patients with nonalcoholic fatty liver disease: a systematic review and meta-analysis. Eur J Gastroenterol Hepatol.

[56] Lu W, Li S, Li J (2016). Effects of omega-3 fatty acid in nonalcoholic fatty liver disease: a meta-analysis. Gastroenterol Res Pract.

[57] Yu L, Yuan M, Wang L (2017). The effect of omega-3 unsaturated fatty acids on non-alcoholic fatty liver disease: a systematic review and meta-analysis of RCTs. Pak J Med Sci.

[58] Musa-Veloso K, Venditti C, Lee HY (2018). Systematic review and meta-analysis of controlled intervention studies on the effectiveness of long-chain omega-3 fatty acids in patients with nonalcoholic fatty liver disease. Nutr Rev.

[59] Smart NA, King N, McFarlane JR, Graham PL, Dieberg G (2018). Effect of exercise training on liver function in adults who are overweight or exhibit fatty liver disease: a systematic review and meta-analysis. Br J Sports Med.

[60] Katsagoni CN, Georgoulis M, Papatheodoridis GV, Panagiotakos DB, Kontogianni MD (2017). Effects of lifestyle interventions on clinical characteristics of patients with non-alcoholic fatty liver disease: a meta-analysis. Metabolism.

[61] Zou TT, Zhang C, Zhou YF (2018). Lifestyle interventions for patients with nonalcoholic fatty liver disease: a network meta-analysis. Eur J Gastroenterol Hepatol.

[62] Yan JH, Guan BJ, Gao HY, Peng XE (2018). Omega-3 polyunsaturated fatty acid supplementation and non-alcoholic fatty liver disease: a meta-analysis of randomized controlled trials. Medicine (Baltimore).

[63] He XX, Wu XL, Chen RP (2016). Effectiveness of omega-3 polyunsaturated fatty acids in non-alcoholic fatty liver disease: a meta-analysis of randomized controlled trials. Plos One.

[64] Guo XF, Yang B, Tang J, Li D (2018). Fatty acid and non-alcoholic fatty liver disease: meta-analyses of case-control and randomized controlled trials. Clin Nutr.

[65] European Association for the Study of the Liver (EASL), European Association for the Study of Diabetes (EASD), European Association for the Study of Obesity (EASO) (2016). EASL-EASD-EASO Clinical Practice Guidelines for the management of non-alcoholic fatty liver disease. J Hepatol.

[66] Fatty liver and Alcoholic Liver Disease Group, Hepatology Branch, Chinese Medical Association, Fatty liver Disease Expert Committee, Chinese Medical Association. Guidelines for the Prevention and treatment of nonalcoholic fatty liver disease (2018). Chin J Liver Dis. 2018;34(5):195–203.

[67] Neuenschwander M, Ballon A, Weber KS (2019). Role of diet in type 2 diabetes incidence: umbrella review of meta-analyses of prospective observational studies. BMJ.

[68] Malik VS, Hu FB (2012). Sweeteners and risk of obesity and type 2 diabetes: the role of sugar-sweetened beverages. Curr Diab Rep..

[69] Atkinson FS, Foster-Powell K, Brand-Miller JC (2008). International tables of glycemic index and glycemic load values: 2008. Diabetes Care.

[70] Barrera F, George J (2014). The role of diet and nutritional intervention for the management of patients with NAFLD. Clin Liver Dis.

[71] Beysen C, Ruddy M, Stoch A (2018). Dose-dependent quantitative effects of acute fructose administration on hepatic de novo lipogenesis in healthy humans. Am J Physiol Endocrinol Metab.

[72] Lambert JE, Ramos-Roman MA, Browning JD, Parks EJ (2014). Increased de novo lipogenesis is a distinct characteristic of individuals with nonalcoholic fatty liver disease. Gastroenterology.

[73] Emamat H, Ghalandari H, Tangestani H, Abdollahi A, Hekmatdoost A (2020). Artificial sweeteners are related to non-alcoholic fatty liver disease: microbiota dysbiosis as a novel potential mechanism. EXCLI J.

[74] Li X, Meng X, Timofeeva M (2017). Serum uric acid levels and multiple health outcomes: umbrella review of evidence from observational studies, randomised controlled trials, and Mendelian randomisation studies. BMJ.

[75] Yamada T, Suzuki S, Fukatsu M, Wada T, Yoshida T, Joh T (2010). Elevated serum uric acid is an independent risk factor for nonalcoholic fatty liver disease in Japanese undergoing a health checkup. Acta Gastroenterol Belg.

[76] Lanaspa MA, Sanchez-Lozada LG, Cicerchi C (2012). Uric acid stimulates fructokinase and accelerates fructose metabolism in the development of fatty liver. Plos One.

[77] Crespo M, Lappe S, Feldstein AE, Alkhouri N (2016). Similarities and differences between pediatric and adult nonalcoholic fatty liver disease. Metabolism.

[78] Paulweber B, Valensi P, Lindström J (2010). A European evidence-based guideline for the prevention of type 2 diabetes. Horm Metab Res.

[79] Poole R, Kennedy OJ, Roderick P, Fallowfield JA, Hayes PC, Parkes J (2017). Coffee consumption and health: umbrella review of meta-analyses of multiple health outcomes. BMJ.

[80] Mayer EJ, Newman B, Quesenberry CP, Friedman GD, Selby JV (1993). Alcohol consumption and insulin concentrations. Role of insulin in associations of alcohol intake with high-density lipoprotein cholesterol and triglycerides. Circulation.

[81] Facchini F, Chen YD, Reaven GM (1994). Light-to-moderate alcohol intake is associated with enhanced insulin sensitivity. Diabetes Care.

[82] Lazarus R, Sparrow D, Weiss ST (1997). Alcohol intake and insulin levels. the normative aging study. Am J Epidemiol.

[83] Kampman E, Thompson RL, Wiseman MJ, Mitrou G, Allen K (2018). PO-087 The wcrf/aicr third expert report on diet, nutrition, physical activity and cancer: updated recommendations.

[84] Keijzers GB, Galan BD, Tack CJ, Smits P (2002). Caffeine can decrease insulin sensitivity in humans. Diabetes Care.

[85] Saab S, Mallam D, Cox GA, Tong MJ (2014). Impact of coffee on liver diseases: a systematic review. Liver Int.

[86] Chen S, Teoh NC, Chitturi S, Farrell GC (2014). Coffee and non-alcoholic fatty liver disease: brewing evidence for hepatoprotection. J Gastroenterol Hepatol.

[87] Yi M, Wu X, Zhuang W (2019). Tea consumption and health outcomes: umbrella review of meta-analyses of observational studies in humans. Mol Nutr Food Res.

[88] Wong VW, Chan WK, Chitturi S (2018). Asia-Pacific Working Party on non-alcoholic fatty liver disease guidelines 2017-Part 1: definition, risk factors and assessment. J Gastroenterol Hepatol.

[89] Chitturi S, Wong VW, Chan WK (2018). The Asia-Pacific Working Party on non-alcoholic fatty liver disease guidelines 2017-Part 2: management and special groups. J Gastroenterol Hepatol.

[90] Grosso G, Godos J, Galvano F, Giovannucci EL (2017). Coffee, caffeine, and health outcomes: an umbrella review. Annu Rev Nutr.

[91] Vos MB, Abrams SH, Ba Rlow SE (2017). NASPGHAN Clinical Practice Guideline for the Diagnosis and Treatment of Nonalcoholic Fatty Liver Disease in Children: Recommendations from the Expert Committee on NAFLD (ECON) and the North American Society of Pediatric Gastroenterology, Hepatology and Nut. J Pediatr Gastroenterol Nutr.

[92] Plauth M, Bernal W, Dasarathy S (2019). ESPEN guideline on clinical nutrition in liver disease - ScienceDirect. Clin Nutr.

[93] Masterjohn C, Bruno RS (2012). Therapeutic potential of green tea in nonalcoholic fatty liver disease. Nutr Rev.

[94] Bruno RS, Dugan CE, Smyth JA, DiNatale DA, Koo SI (2008). Green tea extract protects leptin-deficient, spontaneously obese mice from hepatic steatosis and injury. J Nutr.

[95] Park HJ, DiNatale DA, Chung MY (2011). Green tea extract attenuates hepatic steatosis by decreasing adipose lipogenesis and enhancing hepatic antioxidant defenses in ob/ob mice. J Nutr Biochem.

[96] Pettinelli P, Del Pozo T, Araya J (2009). Enhancement in liver SREBP-1c/PPAR-alpha ratio and steatosis in obese patients: correlations with insulin resistance and n-3 long-chain polyunsaturated fatty acid depletion. Biochim Biophys Acta.

[97] Calder P (2010). Marine omega-3 fatty acids and inflammation. J Lipid Nutr.

[98] Browning JD, Baker JA, Rogers T, Davis J, Satapati S, Burgess SC (2011). Short-term weight loss and hepatic triglyceride reduction: evidence of a metabolic advantage with dietary carbohydrate restriction. Am J Clin Nutr..

[99] Browning JD, Weis B, Davis J (2008). Alterations in hepatic glucose and energy metabolism as a result of calorie and carbohydrate restriction. Hepatology.

