# Supplementary material for: Lifestyle as well as metabolic syndrome and non-alcoholic fatty liver disease: an umbrella review of evidence from observational studies and randomized controlled trials
Source: BMC Endocr Disord. 2022 Apr 10;22:95. doi: 10.1186/s12902-022-01015-5 (PMC8996397; doi:10.1186/s12902-022-01015-5)
Supplement: Supplementary file 1 — Additional file 1. [file 12902_2022_1015_MOESM1_ESM.docx]

**Supplementary Table 1 Evaluation of the methodological quality with AMSTAR**

| **Item** | **Description** |
| --- | --- |
| **Q1** | Was an ‚a priori‘ design provided? |
| **Q2** | Was there duplicate study selection and data extraction? |
| **Q3** | Was a comprehensive literature search performed? |
| **Q4** | Was the status of publication (i.e. grey literature) used as an inclusion criterion? |
| **Q5** | Was a list of studies (included and excluded) provided? |
| **Q6** | Were the characteristics of the included studies provided? |
| **Q7** | Was the scientific quality of the included studies assessed and documented? |
| **Q8** | Was the scientific quality of the included studies used appropriately in formulation conclusions? |
| **Q9** | Were the methods used to combine the findings of studies appropriate? |
| **Q10** | Was the likelihood of publication bias assessed? |
| **Q11** | Was the conflict of interest included? |
| Each question can be answered with “yes,” “no,” “can’t answer,” and “not applicable.” A “yes” scores one point, whereas the other answers score 0 points.  An overall score of at least 8 points was defined as the cutoff value for high quality, 4-7 points as moderate quality, and 3 points or less as low quality. | |

Q = Question
