# Supplementary material for: Lifestyle as well as metabolic syndrome and non-alcoholic fatty liver disease: an umbrella review of evidence from observational studies and randomized controlled trials
Source: BMC Endocr Disord. 2022 Apr 10;22:95. doi: 10.1186/s12902-022-01015-5 (PMC8996397; doi:10.1186/s12902-022-01015-5)
Supplement: Supplementary file 2 — Additional file 2. [file 12902_2022_1015_MOESM2_ESM.docx]

**Supplementary Table** **2** General characteristics and main findings of the meta-analyses of excluded studies

| **Author** | **Year** | **Exposure** | **Measure** | **N**  **Studies** | **N**  **Participants** | **N**  **Cases** | **Type of metric** | **Effect model**  **reported** | **Reported summary effects (95% CI)** | ***p-*value** |
| --- | --- | --- | --- | --- | --- | --- | --- | --- | --- | --- |
| **4 meta-analyses of observational studies** | | | | | | | | | | |
| Marventano^1^ | 2016 | Coffee | NAFLD | 7 | 20620 | 943 | RR | Random | 0.74 (-0.19, 1.56) | 0.03 |
| Wijarnpreecha^2^ | 2016 | Hyperuricemia | NAFLD | NA | NA | NA | OR | Random | 1.97 (1.69, 2.29) | *<*0.00001 |
| Gong^3^ | 2016 | Hyperuricemia | NAFLD | 13 | 117712 | 28446 | RR | Random | 1.79（1.55, 2.07） | <0.001 |
| Zhou^4^ | 2015 | Hyperuricemia | NAFLD | 9 | NA | NA | OR | Random | 1.92 (1.59, 2.31) | 0.001 |
| **18 meta-analyses of RCTs studies** | | | | | | | | | | |
| Chiu^5^ | 2014 | Hypercaloric fructose diet | IHCL | 60 | NA | NA | SMD | Random | 0.45 (0.18, 0.72) | 0.001 |
| He^6^ | 2016 | Omega-3 PUFAs | ALT | 6 | 396 | 197 | IV | Random | −7.61 (−12.83, −2.39) | 0.004 |
| He^6^ | 2016 | Omega-3 PUFAs | AST | 5 | 362 | 180 | IV | Random | −6.89 (−17.71, 3.92) | 0.21 |
| He^6^ | 2016 | Omega-3 PUFAs | GGT | 3 | 181 | 90 | IV | Random | −8.28 (−18.38, 1.83) | 0.11 |
| He^6^ | 2016 | Omega-3 PUFAs | TC | 6 | 396 | 197 | IV | Random | −13.41 (−21.44, −5.38) | 0.001 |
| He^6^ | 2016 | Omega-3 PUFAs | TG | 7 | 442 | 227 | IV | Random | −43.96 (−51.21, −36.71) | *<*0.00001 |
| He^6^ | 2016 | Omega-3 PUFAs | LDL | 5 | 360 | 179 | IV | Random | −7.13 (−14.26, −0.0) | 0.05 |
| He^6^ | 2016 | Omega-3 PUFAs | HDL | 5 | 362 | 180 | IV | Random | 6.97 (2.05, 11.90) | 0.06 |
| Lu^7^ | 2016 | Omega-3 PUFAs | ALT | 8 | 530 | 282 | MD | Random | −4.97 (−11.14, 1.20) | 0.11 |
| Lu^7^ | 2016 | Omega-3 PUFAs | AST | 7 | 496 | 265 | MD | Random | −2.01 (−8.72, 4.70) | 0.56 |
| Lu^7^ | 2016 | Omega-3 PUFAs | GGT | 4 | 237 | 132 | MD | Random | −9.02 (−14.80, −3.24) | 0.002 |
| Lu^7^ | 2016 | Omega-3 PUFAs | HDL | 7 | 472 | 239 | MD | Random | 5.51 (0.03, 11.00) | 0.05 |
| Lu^7^ | 2016 | Omega-3 PUFAs | LDL | 6 | 433 | 220 | MD | Random | 1.28 (−4.06, 6.63) | 0.64 |
| Lu^7^ | 2016 | Omega-3 PUFAs | TG | 9 | 561 | 297 | MD | Random | −35.55 (−53.90, −17.19) | 0.0001 |

**(*Continued*)**

| **Author** | **Year** | **Exposure** | **Measure** | **N**  **Studies** | **N**  **Participants** | **N**  **Cases** | **Type of metric** | **Effect model**  **reported** | **Reported summary effects (95% CI)** | ***p-*value** |
| --- | --- | --- | --- | --- | --- | --- | --- | --- | --- | --- |
| Guo^8^ | 2017 | Omega-3 PUFAs | TG | 11 | 536 | 284 | WMD | Random | -36.16 (-49.15, -23.18) | <0.001 |
| Musa-Veloso^9^ | 2017 | Omega-3 PUFAs | AST | 12 | NA | NA | MD | Random | -2.41 ( -7.35, 2.52) | 0.338 |
| Musa-Veloso^9^ | 2017 | Omega-3 PUFAs | ALT | 16 | NA | NA | MD | Random | -4.63 (-9.18, -0.08) | 0.046 |
| Musa-Veloso^9^ | 2017 | Omega-3 PUFAS | GGT | 8 | NA | NA | MD | Random | -5.56 (-9.61, -1.50) | 0.007 |

IHCL, intrahepatocellular lipids; ALT, alanine aminotransferase; AST, aspartate aminotransferase; TG, triglyceride; TC, total cholesterol; LDL, low-density lipoprotein; GGT, g-glutamyl transferase; HDL, high density lipoprotein; RR, relative risk; OR, odds ratio; MD, mean difference; WMD, weighted mean difference; SMD, standardized mean difference; IV, inverse variance; CI, confidence interval; NA, not available.

**References**

[1] Marventano S, Salomone F, Godos J, et al. Coffee and tea consumption in relation with non-alcoholic fatty liver and metabolic syndrome: A systematic review and meta-analysis of observational studies. Clin Nutr. 2016. 35(6): 1269-1281.

[2] Wijarnpreecha K, Panjawatanan P, Lekuthai N, Thongprayoon C, Cheungpasitporn W, Ungprasert P. Hyperuricaemia and risk of nonalcoholic fatty liver disease: A meta-analysis. Liver Int. 2017. 37(6): 906-918.

[3] Gong S, Song J, Wang L, Zhang S, Wang Y. Hyperuricemia and risk of nonalcoholic fatty liver disease: a systematic review and meta-analysis. Eur J Gastroenterol Hepatol. 2016. 28(2): 132-8.

[4] Zhou Y, Wei F, Fan Y. High serum uric acid and risk of nonalcoholic fatty liver disease: A systematic review and meta-analysis. Clin Biochem. 2016. 49(7-8): 636-42.

[5] Chiu S, Sievenpiper JL, de Souza RJ, et al. Effect of fructose on markers of non-alcoholic fatty liver disease (NAFLD): a systematic review and meta-analysis of controlled feeding trials. Eur J Clin Nutr. 2014. 68(4): 416-23.

[6] He XX, Wu XL, Chen RP, et al. Effectiveness of Omega-3 Polyunsaturated Fatty Acids in Non-Alcoholic Fatty Liver Disease: A Meta-Analysis of Randomized Controlled Trials. PLoS One. 2016. 11(10): e0162368.

[7] Lu W, Li S, Li J, et al. Effects of Omega-3 Fatty Acid in Nonalcoholic Fatty Liver Disease: A Meta-Analysis. Gastroenterol Res Pract. 2016. 2016: 1459790.

[8] Guo XF, Yang B, Tang J, Li D. Fatty acid and non-alcoholic fatty liver disease: Meta-analyses of case-control and randomized controlled trials. Clin Nutr. 2018. 37(1): 113-122.

[9] Musa-Veloso K, Venditti C, Lee HY, et al. Systematic review and meta-analysis of controlled intervention studies on the effectiveness of long-chain omega-3 fatty acids in patients with nonalcoholic fatty liver disease. Nutr Rev. 2018. 76(8): 581-602.
