# Supplementary material for: Lifestyle as well as metabolic syndrome and non-alcoholic fatty liver disease: an umbrella review of evidence from observational studies and randomized controlled trials
Source: BMC Endocr Disord. 2022 Apr 10;22:95. doi: 10.1186/s12902-022-01015-5 (PMC8996397; doi:10.1186/s12902-022-01015-5)
Supplement: Supplementary file 3 — Additional file 3. [file 12902_2022_1015_MOESM3_ESM.docx]

**Supplementary Table 3** General characteristics and main findings of 37 meta-analyses of observational studies

| **Author** | **Year** | **Exposure** | **Measure** | **N**  **Studies** | **N**  **Participants** | **N**  **Cases** | **Type of metric** | **Effect model**  **reported** | **Reported summary effects (95% CI)** | ***p-*value** |
| --- | --- | --- | --- | --- | --- | --- | --- | --- | --- | --- |
| **15 factors that increase the risk of NAFLD** | | | | | | | | | | |
| Rezayat^1^ | 2017 | Smoking | NAFLD | 20 | 92125 | 20149 | OR | Random | 1.43 (1.02, 1.84) | 0.008 |
| Rezayat^1^ | 2017 | Passive smoking | NAFLD | 2 | NA | NA | OR | Random | 1.32 (1.16, 1.50) | 0.001 |
| Rezayat^1^ | 2017 | Former smoking | NAFLD | 4 | 2210 | 784 | OR | Random | 1.38 (1.20, 1.59) | 0.001 |
| Asgar-Taee^2^ | 2018 | Sugar sweetened beverages | NAFLD | 4 | 5241 | 1150 | OR | Random | 1.40 (1.07, 1.82) | 0.013 |
| Wijarnpreecha^3^ | 2015 | Sugar-Sweetened Soda | NAFLD | 7 | 4639 | NA | RR | Random | 1.53 (1.34, 1.75) | <0.00001 |
| He^4^ | 2020 | Soft drinks | NAFLD | 7 | 32788 | 9947 | OR | Random | 1.33 (1.18, 1.49) | 0.000 |
| Chung^5^ | 2014 | Hypercaloric fructose diet | IHCL | 6 | NA | NA | OR | Random | 1.13 (1.02, 1.45) | 0.001 |
| He^4^ | 2020 | Red meat | NAFLD | 8 | NA | 8115 | OR | Random | 1.26 (1.08, 1.47) | 0.002 |
| Wijarnpreecha^6^ | 2016 | Short sleep duration | NAFLD | 6 | 59094 | NA | RR | Random | 1.19 (1.04, 1.36) | 0.011 |
| Li^7^ | 2016 | Obesity | NAFLD | 21 | 381655 | NA | RR | Random | 3.53 (2.48, 5.03) | <0.001 |
| Pang^8^ | 2015 | Per 1-unit increase in WC | NAFLD | 11 | 37941 | 10454 | OR | Random | 1.07 (1.03, 1.10) | 0.000 |
| Pang^8^ | 2015 | Per 1-unit increase in BMI | NAFLD | 11 | 37941 | 10454 | OR | Random | 1.25 (1.13, 1.38) | 0.000 |
| Pang^8^ | 2015 | WHR | NAFLD | 3 | 1063 | 387 | OR | Random | 4.10 (1.53, 10.79) | 0.005 |
| Darmawan^9^ | 2017 | Hyperuricemia | NAFLD | 11 | 100725 | 18303 | OR | Random | 1.92 (1.66, 2.23) | <0.00001 |
| Jaruvongvanich^10^ | 2017 | Hyperuricemia | NAS | 5 | 777 | NA | RR | Random | 2.17 (1.51, 3.12) | <0.001 |
| **7 factors that decrease the risk of NAFLD** | | | | | | | | | | |
| Sookoian^11^ | 2014 | Modest alcohol | NAFLD | 8 | 43175 | 12384 | OR | Random | 0.68 (0.58, 0.81) | <0.00001 |
| Sookoian^11^ | 2014 | Modest alcohol | NASH | 2 | 822 | 272 | OR | Random | 0.50 (0.34, 0.74) | <0.0005 |
| Chen^12^ | 2018 | Coffee | NAFLD | 7 | 54441 | 4825 | RR | Random | 0.94 (0.92, 0.97) | <0.001 |
| Wijarnpreecha^13^ | 2017 | Coffee | Liver fibrosis | 3 | NA | 883 | RR | Random | 0.70 (0.60, 0.82) | <0.00001 |
| Yin^14^ | 2015 | Green tea | Liver steatosis | 4 | 2005 | 600 | RR | Random | 0.65 (0.44, 0.98) | 0.039 |

**(*Continued)***

| **Author** | **Year** | **Exposure** | **Measure** | **N**  **Studies** | **N**  **Participants** | **N**  **Cases** | **Type of metric** | **Effect model**  **reported** | **Reported summary effects (95% CI)** | ***p-*value** |
| --- | --- | --- | --- | --- | --- | --- | --- | --- | --- | --- |
| He^4^ | 2020 | Nut | NAFLD | 5 | NA | 5505 | OR | Random | 0.94 (0.90, 0.97) | 0.001 |
| Koutoukidis^15^ | 2019 | Weight loss | NASH | 2 | 73 | 41 | OR | Random | 0.14 (0.04,0.49) | 0.002 |
| **15 factors that do not affect the risk of NAFLD** | | | | | | | | | | |
| Rezayat^1^ | 2017 | Current smoking | NAFLD | 4 | 1889 | 555 | OR | Random | 1.03 (0.90, 1.19) | >0.05 |
| Rezayat^1^ | 2017 | Light smoking | NAFLD | 5 | 7113 | 2009 | OR | Random | 1.07 (0.99, 1.33) | >0.05 |
| Rezayat^1^ | 2017 | Heavy smoking | NAFLD | 4 | 1682 | NR | OR | Random | 1.01 (0.90, 1.15) | >0.05 |
| He^4^ | 2020 | Whole grains | NAFLD | 3 | NR | 2467 | OR | Random | 1.01 (0.98, 1.04) | >0.05 |
| He^4^ | 2020 | Refined grains | NAFLD | 6 | NR | 3716 | OR | Random | 1.01 (0.95, 1.08) | >0.05 |
| He^4^ | 2020 | Fish | NAFLD | 6 | NR | 2914 | OR | Random | 0.90 (0.73, 1.10) | >0.05 |
| He^4^ | 2020 | Fruits | NAFLD | 8 | 27867 | 14092 | OR | Random | 0.96 (0.84, 1.09) | >0.05 |
| He^4^ | 2020 | Vegetables | NAFLD | 8 | NR | 7597 | OR | Random | 0.97 (0.89, 1.05) | >0.05 |
| He^4^ | 2020 | Eggs | NAFLD | 3 | NR | 2300 | OR | Random | 0.97 (0.82, 1.15) | >0.05 |
| He^4^ | 2020 | Dairy | NAFLD | 4 | NR | 6932 | OR | Random | 1.02 (0.87, 1.18) | >0.05 |
| He^4^ | 2020 | Legumes | NAFLD | 4 | NR | 2810 | OR | Random | 0.83 (0.67, 1.01) | >0.05 |
| Chiu^16^ | 2014 | Hypercaloric fructose diet | ALT | NA | NA | NA | MD | Random | 4.94 (0.03, 9.85) | 0.05 |
| Shen^17^ | 2016 | Caffeine | NAFLD | 3 | 16994 | 1996 | MD | Random | 2.36 (–35.92, 40.64) | 0.90 |
| Ahn^18^ | 2018 | Low carbohydrate diet | ALT | 5 | 165 | 85 | RR | Random | 0.50 (-0.13, 1.14) | 0.12 |
| Ahn^18^ | 2018 | Low carbohydrate diet | AST | 4 | 130 | 67 | RR | Random | 0.61 (-0.18, 1.40) | 0.13 |

NAFLD, nonalcoholic fatty liver disease; IHCL, intrahepatocellular lipids; NAS, non-alcoholic activity score; Omega-3 PUFAs, omega-3 polyunsaturated fatty acids; NASH, non-alcoholic steatohepatitis; ALT, alanine aminotransferase; AST, aspartate aminotransferase; OR, odds ratio; RR, relative risk; MD, mean difference; CI, confidence interval; NA, not available; NR, not report;

**References**

[1] Akhavan Rezayat A, Dadgar Moghadam M, Ghasemi Nour M, et al. Association between smoking and non-alcoholic fatty liver disease: A systematic review and meta-analysis. SAGE Open Med. 2018. 6: 2050312117745223.

[2] Asgari-Taee F, Zerafati-Shoae N, Dehghani M, Sadeghi M, Baradaran HR, Jazayeri S. Association of sugar sweetened beverages consumption with non-alcoholic fatty liver disease: a systematic review and meta-analysis. Eur J Nutr. 2019. 58(5): 1759-1769.

[3] Wijarnpreecha K, Thongprayoon C, Edmonds PJ, Cheungpasitporn W. Associations of sugar- and artificially sweetened soda with nonalcoholic fatty liver disease: a systematic review and meta-analysis. QJM. 2016. 109(7): 461-466.

[4] He K, Li Y, Guo X, Zhong L, Tang S. Food groups and the likelihood of nonalcoholic fatty liver disease: a systematic review and meta-analysis. Br J Nutr. 2020 : 1-40.

[5] Chung M, Ma J, Patel K, Berger S, Lau J, Lichtenstein AH. Fructose, high-fructose corn syrup, sucrose, and nonalcoholic fatty liver disease or indexes of liver health: a systematic review and meta-analysis. Am J Clin Nutr. 2014. 100(3): 833-49.

[6] Wijarnpreecha K, Thongprayoon C, Panjawatanan P, Ungprasert P. Short sleep duration and risk of nonalcoholic fatty liver disease: A systematic review and meta-analysis. J Gastroenterol Hepatol. 2016. 31(11): 1802-1807.

[7] Li L, Liu DW, Yan HY, Wang ZY, Zhao SH, Wang B. Obesity is an independent risk factor for non-alcoholic fatty liver disease: evidence from a meta-analysis of 21 cohort studies. Obes Rev. 2016. 17(6): 510-9.

[8] Pang Q, Zhang JY, Song SD, et al. Central obesity and nonalcoholic fatty liver disease risk after adjusting for body mass index. World J Gastroenterol. 2015. 21(5): 1650-62.

[9] Darmawan G, Hamijoyo L, Hasan I. Association between Serum Uric Acid and Non-Alcoholic Fatty Liver Disease: A Meta-Analysis. Acta Med Indones. 2017. 49(2): 136-147.

[10] Jaruvongvanich V, Ahuja W, Wirunsawanya K, Wijarnpreecha K, Ungprasert P. Hyperuricemia is associated with nonalcoholic fatty liver disease activity score in patients with nonalcoholic fatty liver disease: a systematic review and meta-analysis. Eur J Gastroenterol Hepatol. 2017. 29(9): 1031-1035.

[11] Sookoian S, Castaño GO, Pirola CJ. Modest alcohol consumption decreases the risk of non-alcoholic fatty liver disease: a meta-analysis of 43 175 individuals. Gut. 2014. 63(3): 530-2.

[12] Chen YP, Lu FB, Hu YB, Xu LM, Zheng MH, Hu ED. A systematic review and a dose-response meta-analysis of coffee dose and nonalcoholic fatty liver disease. Clin Nutr. 2019. 38(6): 2552-2557.

[13] Wijarnpreecha K, Thongprayoon C, Ungprasert P. Coffee consumption and risk of nonalcoholic fatty liver disease: a systematic review and meta-analysis. Eur J Gastroenterol Hepatol. 2017. 29(2): e8-e12.

[14] Yin X, Yang J, Li T, et al. The effect of green tea intake on risk of liver disease: a meta analysis. Int J Clin Exp Med. 2015. 8(6): 8339-46.

[15] Koutoukidis DA, Astbury NM, Tudor KE, et al. Association of Weight Loss Interventions With Changes in Biomarkers of Nonalcoholic Fatty Liver Disease: A Systematic Review and Meta-analysis. JAMA Intern Med. 2019 .

[16] Chiu S, Sievenpiper JL, de Souza RJ, et al. Effect of fructose on markers of non-alcoholic fatty liver disease (NAFLD): a systematic review and meta-analysis of controlled feeding trials. Eur J Clin Nutr. 2014. 68(4): 416-23.

[17] Shen H, Rodriguez AC, Shiani A, et al. Association between caffeine consumption and nonalcoholic fatty liver disease: a systemic review and meta-analysis. Therap Adv Gastroenterol. 2016. 9(1): 113-20.

[18] Ahn J, Jun DW, Lee HY, Moon JH. Critical appraisal for low-carbohydrate diet in nonalcoholic fatty liver disease: Review and meta-analyses. Clin Nutr. 2019. 38(5): 2023-2030.
