# Supplementary material for: Lifestyle as well as metabolic syndrome and non-alcoholic fatty liver disease: an umbrella review of evidence from observational studies and randomized controlled trials
Source: BMC Endocr Disord. 2022 Apr 10;22:95. doi: 10.1186/s12902-022-01015-5 (PMC8996397; doi:10.1186/s12902-022-01015-5)
Supplement: Supplementary file 4 — Additional file 4. [file 12902_2022_1015_MOESM4_ESM.docx]

**Supplementary Table 4** General characteristics and main findings of 63 meta-analyses of RCTs

| **Author** | **Year** | **Exposure** | **Measure** | **N**  **Studies** | **N**  **Participants** | **N**  **Cases** | **Type of metric** | **Effect model**  **reported** | **Reported summary effects (95% CI)** | ***p-*value** |
| --- | --- | --- | --- | --- | --- | --- | --- | --- | --- | --- |
| **Therapies that improve NAFLD** | | | | | | | | | | |
| Shen^1^ | 2016 | Caffeine | liver fibrosis | 2 | NR | 292 | MD | Random | −91.35 (−139.42, −43.27) | 0.0002 |
| Mansour-Ghanaei^2^ | 2018 | Green tea | ALT | 4 | 234 | 122 | MD | Random | -12.81 (-18.17, -7.45) | <0.00001 |
| Mansour-Ghanaei^2^ | 2018 | Green tea | AST | 4 | 234 | 122 | MD | Random | -10.91 (-19.66, -2.17) | 0.01 |
| Mansour-Ghanaei^2^ | 2018 | Green tea | TG | 3 | 163 | 87 | MD | Random | -31.86 (-40.62, -23.12) | <0.00001 |
| Mansour-Ghanaei^2^ | 2018 | Green tea | TC | 3 | 163 | 87 | MD | Random | -27.57 (-36.17, -18.98) | <0.00001 |
| Mansour-Ghanaei^2^ | 2018 | Green tea | LDL | 3 | 163 | 87 | MD | Random | -14.15 (-23.69, -4.60) | 0.004 |
| Mansour-Ghanaei^2^ | 2018 | Green tea | BMI | 4 | 234 | 122 | MD | Random | -2.08 (-2.81, -1.36) | <0.00001 |
| Haghighatdoost^3^ | 2016 | Low carbohydrate diet | IHCL | 4 | NA | 238 | Mean  percentage | Random | -11.53% (-18.10, -4.96) | 0.00085 |
| Yan^4^ | 2018 | Omega-3 PUFAs | ALT | 14 | 937 | NA | SMD | Random | -0.50 (-0.88, -0.11) | 0.000 |
| Yan^4^ | 2018 | Omega-3 PUFAs | AST | 12 | 903 | NA | SMD | Random | -0.54 (-1.04, -0.05) | 0.000 |
| Yan^4^ | 2018 | Omega-3 PUFAs | GGT | 8 | 1121 | NA | SMD | Random | -0.48 (-0.64, -0.31) | 0.013 |
| Yan^4^ | 2018 | Omega-3 PUFAs | HOMR-IR | 8 | 502 | NA | SMD | Random | -0.40 (-0.58, -0.22) | 0.001 |
| Yan^4^ | 2018 | Omega-3 PUFAs | Glucose | 8 | 474 | NA | SMD | Random | -0.25 (-0.43, -0.06) | 0.002 |
| Musa-Veloso^5^ | 2017 | Omega-3 PUFAs | Liver fat content | 5 | NA | NA | MD | Random | -5.19% (-9.58%, -0.97%) | 0.021 |
| Musa-Veloso^5^ | 2017 | Omega-3 PUFAs | Grade of steatosis | 7 | NA | NA | MD | Random | -0.71 (-0.99, -0.42) | <0.001 |
| Parker^6^ | 2012 | Omega-3 PUFAs | liver fat | 7 | NA | 355 | ES | Random | -0.97 (-0.58, -1.35) | <0.001 |
| Yan^4^ | 2018 | Omega-3 PUFAs | TG | 16 | 1075 | NA | SMD | Random | -0.47 (-0.76, -0.19) | 0.002 |

**(*Continued)***

| **Author** | **Year** | **Exposure** | **Measure** | **N**  **Studies** | **N**  **Participants** | **N**  **Cases** | **Type of metric** | **Effect model**  **reported** | **Reported summary effects (95% CI)** | ***p-*value** |
| --- | --- | --- | --- | --- | --- | --- | --- | --- | --- | --- |
| Yu^7^ | 2017 | Omega-3 PUFAs | LDL | 6 | 468 | 235 | MD | Random | -9.18 (-14.89, -3.47) | 0.002 |
| Yu^7^ | 2017 | Omega-3 PUFAs | HDL | 7 | 509 | 254 | MD | Random | 4.81 (1.59, 8.03) | 0.03 |
| Smart^8^ | 2016 | Total exercise | Intrahepatic fat | 21 | 1530 | NA | SMD | Random | -1.77 (-3.11, -0.42) | 0.01 |
| Katsagoni^9^ | 2016 | Total exercise (irrespectively of  weight change) | IHTG | 10 | 540 | 325 | SMD | Random | -0.98 (-1.30, -0.66) | <0.001 |
| Katsagoni^9^ | 2016 | Total exercise (irrespectively of  weight change) | ALT | 11 | 495 | 301 | SMD | Random | -0.39 (-0.66, -0.11) | 0.006 |
| Katsagoni^9^ | 2016 | Total exercise (irrespectively of  weight change) | AST | 9 | 494 | 373 | SMD | Random | -0.37 (-0.65, -0.09) | 0.009 |
| Katsagoni^9^ | 2016 | Total exercise (irrespectively of  weight change) | WC | NA | 564 | NA | SMD | Random | -0.60 (-0.78, -0.42) | <0.001 |
| Katsagoni^9^ | 2016 | Total exercise (irrespectively of  weight change) | HOMA-IR | NA | 564 | NA | SMD | Random | -0.76 (-1.47, -0.05) | <0.001 |
| Keating^10^ | 2012 | Total exercise (irrespectively of  weight change) | liver fat | 6 | 156 | 93 | ES | Random | -0.37 (-0.69, -0.06) | 0.02 |
| Katsagoni^9^ | 2016 | Exercise(AEx) | IHTG | 5 | 119 | 68 | SMD | Random | -0.84 (-1.27, -0.42) | <0.001 |

**(*Continued)***

| **Author** | **Year** | **Exposure** | **Measure** | **N**  **Studies** | **N**  **Participants** | **N**  **Cases** | **Type of metric** | **Effect model**  **reported** | **Reported summary effects (95% CI)** | ***p-*value** |
| --- | --- | --- | --- | --- | --- | --- | --- | --- | --- | --- |
| Katsagoni^9^ | 2016 | Exercise(RT) | IHTG | 3 | 133 | 72 | SMD | Random | -1.05 (-1.87, -0.24) | 0.011 |
| Katsagoni^9^ | 2016 | Exercise (AEx+ RT) | IHTG | 3 | 61 | 36 | SMD | Random | -1.54 (-2.56, -0.52) | 0.003 |
| Katsagoni^9^ | 2016 | Exercise (continuous MIT) | IHTG | 2 | 229 | 93 | SMD | Random | -0.86 (-1.36, -0.34) | 0.001 |
| Katsagoni^9^ | 2016 | Exercise (L to M volume MIT) | IHTG | 4 | 234 | 124 | SMD | Random | -0.50 (-0.77, -0.23) | <0.001 |
| Zou^11^ | 2018 | Exercise(AEx) | ALT | 20 | 846 | 134 | WMD | Random | -17.04 (−38.08, –4.00) | 0.01 |
| Zou^11^ | 2018 | Exercise(RT) | ALT | 20 | 846 | 71 | WMD | Random | -17.33 (− 43.90, –8.22) | <0.001 |
| Zou^11^ | 2018 | Exercise (AEx+ RT) | ALT | 20 | 846 | 26 | WMD | Random | -32.12 (− 66.11, –1.87) | <0.001 |
| Zou^11^ | 2018 | Exercise(AEx) | AST | 17 | 790 | 110 | WMD | Random | −5.83 (−12.21, –0.45) | <0.001 |
| Zou^11^ | 2018 | Exercise(RT) | AST | 17 | 790 | 60 | WMD | Random | -4.38 (−20.58, 11.83) | <0.001 |
| Zou^11^ | 2018 | Exercise(AEx) | HOMR-IR | 11 | 492 | 69 | WMD | Random | -0.17 (−0.69, 0.36) | <0.001 |
| Zou^11^ | 2018 | Exercise(RT) | HOMR-IR | 11 | 492 | 11 | WMD | Random | -1.70 (− 5.61, 2.21) | <0.001 |
| Zou^11^ | 2018 | Exercise (AEx+ RT) | HOMR-IR | 11 | 492 | 26 | WMD | Random | -0.52 (−1.51, 0.41) | <0.001 |
| Zou^11^ | 2018 | Exercise(AEx) | BMI | 20 | 13426 | 846 | WMD | Random | -1.55 (− 3.52, –0.42) | <0.001 |
| Zou^11^ | 2018 | Exercise(RT) | BMI | 20 | 846 | 71 | WMD | Random | -1.81 (−3.80, -0.18) | <0.001 |
| Zou^11^ | 2018 | Exercise (AEx+ RT) | BMI | 20 | 846 | 26 | WMD | Random | -2.09 (−4.07, –0.10) | <0.001 |
| Koutoukidis^12^ | 2019 | Weight loss | ALT | 21 | 2558 | 1496 | MD | Random | -9.18 (-13.12, -6.50) | <0.001 |
| Koutoukidis^12^ | 2019 | Weight loss | AST | 19 | 2558 | 1446 | MD | Random | -4.84 (-7.13, -2.38) | <0.00001 |
| Koutoukidis^12^ | 2019 | Weight loss | GGT | 9 | 1774 | 1124 | MD | Random | -4.35 (-7.67, -1.04) | <0.00001 |
| Koutoukidis^12^ | 2019 | Weight loss | liver stiffness | 4 | 271 | 151 | SMD | Random | -1.11, (-1.91, -0.32) | <0.00001 |
| Koutoukidis^12^ | 2019 | Weight loss | Liver steatosis | 11 | 765 | 405 | SMD | Random | -1.48 (-2.27, -0.70) | <0.01 |

**(*Continued)***

| **Author** | **Year** | **Exposure** | **Measure** | **N**  **Studies** | **N**  **Participants** | **N**  **Cases** | **Type of metric** | **Effect model**  **reported** | **Reported summary effects (95% CI)** | ***p-*value** |
| --- | --- | --- | --- | --- | --- | --- | --- | --- | --- | --- |
| Koutoukidis^12^ | 2019 | Weight loss | NAS | 5 | 164 | 93 | SMD | Random | -0.92 (-1.75, -0.09) | <0.001 |
| **Therapies that do not significantly improve NAFLD** | | | | | | | | | | |
| Lu^13^ | 2016 | Omega-3 PUFAs | TC | 7 | 474 | 240 | MD | Random | −3.65 (−10.40, 3.09) | 0.29 |
| Katsagoni^9^ | 2016 | Total exercise (irrespectively of  weight change) | GGT | 7 | 446 | 272 | SMD | Random | -0.16 (-0.33, 0.02) | 0.08 |
| Katsagoni^9^ | 2016 | Exercise(AEx) | ALT | 5 | 119 | 68 | SMD | Random | -0.36 (-0.73, -0.001) | 0.05 |
| Katsagoni^9^ | 2016 | Exercise(AEx) | AST | 5 | 119 | 68 | SMD | Random | -0.34 (-0.71, 0.03) | 0.07 |
| Katsagoni^9^ | 2016 | Exercise(AEx) | GGT | 5 | 119 | 68 | SMD | Random | -0.03 (-0.24, 0.17) | 0.73 |
| Katsagoni^9^ | 2016 | Exercise(RT) | ALT | NA | NA | NA | NA | NA | NA | NA |
| Katsagoni^9^ | 2016 | Exercise(RT) | AST | NA | NA | NA | NA | NA | NA | NA |
| Katsagoni^9^ | 2016 | Exercise(RT) | GGT | NA | NA | NA | NA | NA | NA | NA |
| Katsagoni^9^ | 2016 | Exercise(AEx+RT) | ALT | NA | NA | NA | NA | NA | NA | NA |
| Katsagoni^9^ | 2016 | Exercise(AEx+RT) | AST | NA | NA | NA | NA | NA | NA | NA |
| Katsagoni^9^ | 2016 | Exercise(AEx+RT) | GGT | NA | NA | NA | NA | NA | NA | NA |
| Koutoukidis^12^ | 2019 | Weight loss | ALP | 3 | 186 | 96 | MD | Random | -1.11 (-1.91, -0.32) | 0.46 |
| Koutoukidis^12^ | 2019 | Weight loss | Inflammation | 4 | 132 | 72 | MD | Random | -0.01 (-0.10, 0.07) | 0.75 |
| Koutoukidis^12^ | 2019 | Weight loss | Ballooning | 4 | 132 | 72 | MD | Random | -0.11 (-0.26, 0.04) | 0.14 |
| Koutoukidis^12^ | 2019 | Weight loss | Liver fibrosis | 6 | 145 | 81 | MD | Random | -0.13 (-0.54, 0.27) | 0.52 |

IHCL, intrahepatocellular lipids; WHR, waist-tohip ratio; WC, waist circumference; BMI, Body mass index; Omega-3 PUFAs, omega-3 polyunsaturated fatty acids; NASH, non-alcoholic steatohepatitis; NAS, nonalcoholic activity score; ALT, alanine aminotransferase; AST, aspartate aminotransferase; TG, triglyceride;

TC, total cholesterol; LDL, low-density lipoprotein cholesterol; BMI, body mass index; IHCL, intrahepatocellular lipids; GGT, g-glutamyl transferase; HOMA

-IR, homeostasis model assessment of insulin resistance; HDL, high density lipoprotein; AEx, aerobic exercise training; RT, resistance training; MIT: moderate-intensity; HIT, high-intensity training; HIIT, high-intensity interval training; IHTG, intrahepatic triglyceride; MIT: moderate-intensity; ALP, alkaline phosphatase; MD, mean difference; SMD, standardized mean difference; WMD, weighted mean difference; ES, effect size; CI, confidence interval; NA, not available.

**References**

[1] Shen H, Rodriguez AC, Shiani A, et al. Association between caffeine consumption and nonalcoholic fatty liver disease: a systemic review and meta-analysis. Therap Adv Gastroenterol. 2016. 9(1): 113-20.

[2] Mansour-Ghanaei F, Hadi A, Pourmasoumi M, Joukar F, Golpour S, Najafgholizadeh A. Green tea as a safe alternative approach for nonalcoholic fatty liver treatment: A systematic review and meta-analysis of clinical trials. Phytother Res. 2018. 32(10): 1876-1884.

[3] Haghighatdoost F, Salehi-Abargouei A, Surkan PJ, Azadbakht L. The effects of low carbohydrate diets on liver function tests in nonalcoholic fatty liver disease: A systematic review and meta-analysis of clinical trials. J Res Med Sci. 2016. 21: 53.

[4] Yan JH, Guan BJ, Gao HY, Peng XE. Omega-3 polyunsaturated fatty acid supplementation and non-alcoholic fatty liver disease: A meta-analysis of randomized controlled trials. Medicine (Baltimore). 2018. 97(37): e12271.

[5] Musa-Veloso K, Venditti C, Lee HY, et al. Systematic review and meta-analysis of controlled intervention studies on the effectiveness of long-chain omega-3 fatty acids in patients with nonalcoholic fatty liver disease. Nutr Rev. 2018. 76(8): 581-602.

[6] Parker HM, Johnson NA, Burdon CA, Cohn JS, O'Connor HT, George J. Omega-3 supplementation and non-alcoholic fatty liver disease: a systematic review and meta-analysis. J Hepatol. 2012. 56(4): 944-51.

[7] Yu L, Yuan M, Wang L. The effect of omega-3 unsaturated fatty acids on non-alcoholic fatty liver disease: A systematic review and meta-analysis of RCTs. Pak J Med Sci. 2017. 33(4): 1022-1028.

[8] Smart NA, King N, McFarlane JR, Graham PL, Dieberg G. Effect of exercise training on liver function in adults who are overweight or exhibit fatty liver disease: a systematic review and meta-analysis. Br J Sports Med. 2018. 52(13): 834-843.

[9] Katsagoni CN, Georgoulis M, Papatheodoridis GV, Panagiotakos DB, Kontogianni MD. Effects of lifestyle interventions on clinical characteristics of patients with non-alcoholic fatty liver disease: A meta-analysis. Metabolism. 2017. 68: 119-132.

[10] Keating SE, Hackett DA, George J, Johnson NA. Exercise and non-alcoholic fatty liver disease: a systematic review and meta-analysis. J Hepatol. 2012. 57(1): 157-66.

[11] Zou TT, Zhang C, Zhou YF, et al. Lifestyle interventions for patients with nonalcoholic fatty liver disease: a network meta-analysis. Eur J Gastroenterol Hepatol. 2018. 30(7): 747-755.

[12] Koutoukidis DA, Astbury NM, Tudor KE, et al. Association of Weight Loss Interventions With Changes in Biomarkers of Nonalcoholic Fatty Liver Disease: A Systematic Review and Meta-analysis. JAMA Intern Med. 2019 .

[13] Lu W, Li S, Li J, et al. Effects of Omega-3 Fatty Acid in Nonalcoholic Fatty Liver Disease: A Meta-Analysis. Gastroenterol Res Pract. 2016. 2016: 1459790.
