# Supplementary material for: Lifestyle as well as metabolic syndrome and non-alcoholic fatty liver disease: an umbrella review of evidence from observational studies and randomized controlled trials
Source: BMC Endocr Disord. 2022 Apr 10;22:95. doi: 10.1186/s12902-022-01015-5 (PMC8996397; doi:10.1186/s12902-022-01015-5)
Supplement: Supplementary file 5 — Additional file 5. [file 12902_2022_1015_MOESM5_ESM.docx]

**Supplementary Table 5 Evaluation of the methodological quality with AMSTAR**

| **Author** | **Year** | **Exposure** | **Measure** | **All** |
| --- | --- | --- | --- | --- |
| **15 factors that increase the risk of NAFLD** | | | | |
| Rezayat | 2017 | Smoking | NAFLD | 8 |
| Rezayat | 2017 | Passive smoking | NAFLD | 8 |
| Rezayat | 2017 | former smoking | NAFLD | 8 |
| Asgar-Taee | 2018 | Sugar sweetened beverages | NAFLD | 8 |
| Wijarnpreecha | 2015 | Sugar-Sweetened Soda | NAFLD | 9 |
| He | 2020 | Soft drinks | NAFLD | 9 |
| Chung | 2014 | Hypercaloric fructose diet | IHLC | 8 |
| He | 2020 | Red meat | NAFLD | 9 |
| Wijarnpreecha | 2016 | Short sleep duration | NAFLD | 8 |
| Li | 2016 | Obesity | NAFLD | 8 |
| Pang | 2015 | Per 1-unit increase in WC | NAFLD | 8 |
| Pang | 2015 | Per 1-unit increase in BMI | NAFLD | 8 |
| Pang | 2015 | WHR | NAFLD | 8 |
| Darmawan | 2017 | Hyperuricemia | NAFLD | 8 |
| Jaruvongvanich | 2017 | Hyperuricemia | NAS | 8 |
| **7 factors that decrease the risk of NAFLD** | | | | |
| Sookoian | 2014 | Modest alcohol | NAFLD | 8 |
| Sookoian | 2014 | Modest alcohol | NASH | 8 |
| Chen | 2018 | Coffee | NAFLD | 8 |
| Wijarnpreecha | 2017 | Coffee | liver fibrosis | 7 |
| Yin | 2015 | Green tea | liver steatosis | 8 |
| He | 2020 | Nut | NAFLD | 9 |
| Koutoukidis | 2019 | Weight loss | NASH | 9 |
| **15 factors that do not affect the risk of NAFLD** | | | | |
| Rezayat | 2017 | Current smoking | NAFLD | 8 |
| Rezayat | 2017 | Light smoking | NAFLD | 8 |
| Rezayat | 2017 | Heavy smoking | NAFLD | 8 |
| He | 2020 | Whole grains | NAFLD | 9 |
| He | 2020 | Refined grains | NAFLD | 9 |
| He | 2020 | Fish | NAFLD | 9 |
| He | 2020 | Fruits | NAFLD | 9 |
| He | 2020 | Vegetables | NAFLD | 9 |
| He | 2020 | Eggs | NAFLD | 9 |
| He | 2020 | Dairy | NAFLD | 9 |
| He | 2020 | Legumes | NAFLD | 9 |
| Chiu | 2014 | Hypercaloric fructose diet | ALT | 8 |
| Shen | 2016 | Caffeine | NAFLD | 8 |
| Ahn | 2018 | low carbohydrate diet | ALT | 8 |
| Ahn | 2018 | low carbohydrate diet | AST | 8 |

**(*Continued)***

| Author | Year | Exposure | Measure | All |
| --- | --- | --- | --- | --- |
| **Therapies that improve NAFLD** | | | | |
| Shen | 2016 | Caffeine | liver fibrosis | 8 |
| Mansour-Ghanaei | 2018 | Green tea | ALT | 8 |
| Mansour-Ghanaei | 2018 | Green tea | AST | 8 |
| Mansour-Ghanaei | 2018 | Green tea | TG | 8 |
| Mansour-Ghanaei | 2018 | Green tea | TC | 8 |
| Mansour-Ghanaei | 2018 | Green tea | LDL | 8 |
| Mansour-Ghanaei | 2018 | Green tea | BMI | 8 |
| Haghighatdoost | 2016 | Low carbohydrate diet | IHLC | 8 |
| Yan | 2018 | Omega-3 PUFAs | ALT | 9 |
| Yan | 2018 | Omega-3 PUFAs | AST | 9 |
| Yan | 2018 | Omega-3 PUFAs | GGT | 9 |
| Yan | 2018 | Omega-3 PUFAs | HOMR-IR | 9 |
| Yan | 2018 | Omega-3 PUFAs | Glucose | 9 |
| Musa-Veloso | 2017 | Omega-3 PUFAs | Liver fat content | 8 |
| Musa-Veloso | 2017 | Omega-3 PUFAs | Grade of steatosis | 8 |
| Parker | 2012 | Omega-3 PUFAs | liver fat | 8 |
| Yan | 2018 | Omega-3 PUFAs | TG | 9 |
| Yu | 2017 | Omega-3 PUFAs | LDL | 7 |
| Yu | 2017 | Omega-3 PUFAs | HDL | 7 |
| Smart | 2016 | Total exercise | Intrahepatic fat | 8 |
| Katsagoni | 2016 | Total exercise (irrespectively of  weight change) | IHTG | 8 |
| Katsagoni | 2016 | Total exercise (irrespectively of  weight change) | ALT | 8 |
| Katsagoni | 2016 | Total exercise (irrespectively of  weight change) | AST | 8 |
| Katsagoni | 2016 | Total exercise (irrespectively of  weight change) | WC | 8 |
| Katsagoni | 2016 | Total exercise (irrespectively of  weight change) | HOMA-IR | 8 |
| Keating | 2012 | Total exercise (no significant weight loss) | liver fat | 8 |
| Katsagoni | 2016 | Exercise (AEx) | IHTG | 8 |
| Katsagoni | 2016 | Exercise (RT) | IHTG | 8 |
| Katsagoni | 2016 | Exercise (AEx+ RT) | IHTG | 8 |
| Katsagoni | 2016 | Exercise (continuous MIT) | IHTG | 8 |
| Katsagoni | 2016 | Exercise (L to M volume MIT) | IHTG | 8 |
| Zou | 2018 | Exercise (AEx) | ALT | 8 |
| Zou | 2018 | Exercise (RT) | ALT | 8 |
| Zou | 2018 | Exercise (AEx+ RT) | ALT | 8 |

**(*Continued)***

| **Author** | **Year** | **Exposure** | **Measure** | **All** |
| --- | --- | --- | --- | --- |
| Zou | 2018 | Exercise (AEx) | AST | 8 |
| Zou | 2018 | Exercise (RT) | AST | 8 |
| Zou | 2018 | Exercise (AEx) | HOMR-IR | 8 |
| Zou | 2018 | Exercise (RT) | HOMR-IR | 8 |
| Zou | 2018 | Exercise (AEx+ RT) | HOMR-IR | 8 |
| Zou | 2018 | Exercise (AEx) | BMI | 8 |
| Zou | 2018 | Exercise (RT) | BMI | 8 |
| Zou | 2018 | Exercise (AEx+ RT) | BMI | 8 |
| Koutoukidis | 2019 | Weight loss | ALT | 9 |
| Koutoukidis | 2019 | Weight loss | AST | 9 |
| Koutoukidis | 2019 | Weight loss | GGT | 9 |
| Koutoukidis | 2019 | Weight loss | liver stiffness | 9 |
| Koutoukidis | 2019 | Weight loss | Liver steatosis | 9 |
| Koutoukidis | 2019 | Weight loss | NAS | 9 |
| **Therapies that do not significantly improve NAFLD** | | | | |
| Lu | 2016 | Omega-3 PUFAs | TC | 8 |
| Katsagoni | 2016 | Total exercise (irrespectively of  weight change) | GGT | 8 |
| Katsagoni | 2016 | Exercise (AEx) | ALT | 8 |
| Katsagoni | 2016 | Exercise (AEx) | AST | 8 |
| Katsagoni | 2016 | Exercise (AEx) | GGT | 8 |
| Katsagoni | 2016 | Exercise (RT) | ALT | 8 |
| Katsagoni | 2016 | Exercise (RT) | AST | 8 |
| Katsagoni | 2016 | Exercise (RT) | GGT | 8 |
| Katsagoni | 2016 | Exercise (AEx+RT) | ALT | 8 |
| Katsagoni | 2016 | Exercise (AEx+RT) | AST | 8 |
| Katsagoni | 2016 | Exercise (AEx+RT) | GGT | 8 |
| Koutoukidis | 2019 | Weight loss | ALP | 9 |
| Koutoukidis | 2019 | Weight loss | inflammation | 9 |
| Koutoukidis | 2019 | Weight loss | ballooning | 9 |
| Koutoukidis | 2019 | Weight loss | liver fibrosis | 9 |
