# Supplementary material for: Lifestyle as well as metabolic syndrome and non-alcoholic fatty liver disease: an umbrella review of evidence from observational studies and randomized controlled trials
Source: BMC Endocr Disord. 2022 Apr 10;22:95. doi: 10.1186/s12902-022-01015-5 (PMC8996397; doi:10.1186/s12902-022-01015-5)
Supplement: Supplementary file 6 — Additional file 6. [file 12902_2022_1015_MOESM6_ESM.docx]

**Supplementary Table 6** **Characteristics of 15 meta-analyses of RCTs on therapies that do not significantly improve NAFLD**

| **Exposure** | **Author, year** | **Measure** | **Studies**  **(n)** | **Subjects (n)** | **Cases**  **(n)** |  | **Random-effect model**  **Effect size (95% CI)** | **p-value** | **I^2^**  **^(%)^** | **Heterogeneity**  **p-value** | **Small-study effects**  **p-value** |
| --- | --- | --- | --- | --- | --- | --- | --- | --- | --- | --- | --- |
| Omega-3 PUFAs | Yan2018 | TC | 15 | 1424 | NA | SMD | -0.09 (-0.50, 0.33) | >0.05 | 90.3 | <0.001 | NA |
| Total exercise (irrespectively  of weight change) | Katsagoni 2016 | GGT | 7 | 446 | 272 | SMD | -0.16 (-0.33, 0.02) | 0.08 | 39.6 | 0.12 | NA |
| Exercise (AEx) | Katsagoni2016 | ALT | 5 | 119 | 68 | SMD | -0.36 (-0.73, -0.001) | 0.06 | 58.4 | 0.025 | 0.015 |
| Exercise (AEx) | Katsagoni2016 | AST | 5 | 119 | 68 | SMD | -0.34 (-0.71, 0.03) | 0.07 | 61.4 | NA | 0.016 |
| Exercis e(AEx) | Katsagoni2016 | GGT | 5 | 119 | 68 | SMD | -0.03 (-0.24, 0.17) | 0.73 | 0 | NA | NA |
| Exercise (RT) | Katsagoni2016 | ALT | NA | NA | NA | NA | NA | NA | NA | 0.31 | NA |
| Exercise (RT) | Katsagoni2016 | AST | NA | NA | NA | NA | NA | NA | NA | 0.43 | NA |
| Exercise (RT) | Katsagoni2016 | GGT | NA | NA | NA | NA | NA | NA | NA | NA | NA |
| Exercise (AEx+RT) | Katsagoni2016 | ALT | NA | NA | NA | NA | NA | NA | NA | 0.45 | NA |
| Exercise (AEx+RT) | Katsagoni2016 | AST | NA | NA | NA | NA | NA | NA | NA | 0.42 | NA |
| Exercise (AEx+RT) | Katsagoni2016 | GGT | NA | NA | NA | NA | NA | NA | NA | 0.17 | NA |
| Weight loss | Koutoukidis2019 | ALP | 3 | 186 | 96 | MD | -5.53 (-20.48, 9.22) | 0.46 | 96 | <0.00001 | NA |
| Weight loss | Koutoukidis2019 | Inflammation | 4 | 132 | 72 | MD | -0.01 (-0.1, 0.07) | 0.75 | 0 | 0.89 | NA |
| Weight loss | Koutoukidis2019 | Ballooning | 4 | 132 | 72 | MD | -0.11 (-0.26, 0.04) | 0.14 | 43 | 0.15 | NA |
| Weight loss | Koutoukidis2019 | Liver fibrosis | 6 | 145 | 81 | MD | -0.13 (-0.54,0.27) | 0.52 | 68 | 0.009 | NA |

Omega-3 PUFAs, omega-3 polyunsaturated fatty acids; TC, total cholesterol; GGT, g-glutamyl transferase; ALT, alanine aminotransferase; AST, aspartate aminotransferase; ALP, alkaline phosphatase; AEx, aerobic exercise training; RT, resistance training; SMD, standardized mean difference; MD, mean difference; CI, confidence interval; NA,not available.
