# Supplementary material for: Lifestyle as well as metabolic syndrome and non-alcoholic fatty liver disease: an umbrella review of evidence from observational studies and randomized controlled trials
Source: BMC Endocr Disord. 2022 Apr 10;22:95. doi: 10.1186/s12902-022-01015-5 (PMC8996397; doi:10.1186/s12902-022-01015-5)
Supplement: Supplementary file 7 — Additional file 7. [file 12902_2022_1015_MOESM7_ESM.docx]

**Supplementary Table 7 The strength of epidemiologic evidence of 15 meta-analyses of observational studies that did not affect the risk of NAFLD**

| **Exposure** | **Measure** | **Reference** | **Precision of the estimate** | | **Consistency of results** | **No evidence of small-study effects** |
| --- | --- | --- | --- | --- | --- | --- |
|  |  |  | **>1000 disease cases** | **P<0.001** | **I^2^ < 50% and Cochran Q test P > .10** | **P>0.1** |
| Current smoking | NAFLD | Rezayat2017 | No | No | Yes | Yes |
| Light smoking | NAFLD | Rezayat2017 | Yes | No | Yes | Yes |
| Heavy smoking | NAFLD | Rezayat2017 | No | No | No | Yes |
| Whole grains | NAFLD | He2020 | Yes | No | Yes | No |
| Refined grains | NAFLD | He2020 | Yes | No | No | Yes |
| Fish | NAFLD | He2020 | Yes | No | No | Yes |
| Fruits | IHCL | He2020 | Yes | No | No | Yes |
| Vegetables | NAFLD | He2020 | Yes | No | Yes | No |
| Eggs | NAFLD | He2020 | Yes | No | No | Yes |
| Dairy | NAFLD | He2020 | Yes | No | No | Yes |
| Legumes | NAFLD | He2020 | Yes | No | No | Yes |
| Hypercaloric fructose diet | ALT | Chiu2014 | No | No | No | Yes |
| Caffeine | NAFLD | Shen2016 | Yes | No | No | Yes |
| Low carbohydrate diet | ALT | Ahn2018 | No | No | No | No |
| Low carbohydrate diet | AST | Ahn2018 | No | No | No | No |

NAFLD, nonalcoholic fatty liver disease; IHCL, intrahepatocellular lipids; ALT, alanine aminotransferase; AST, aspartate aminotransferase;

**NOTE. The strength of epidemiologic evidence was rated as follows:**
High, if all criteria were satisfied: precision of the estimate (P < .001 and >1000 disease cases), consistency of results (I^2^ < 50% and Cochran Q test P > .10), and no evidence of smallstudy effects (P > .10).
Moderate, if a maximum of 1 criterion was not satisfied and a P < .001 was found.
Low, in other cases (P < .05).
